# Computational and Experimental Ballistic Behavior of Epoxy Composites Reinforced with Carnauba Fibers: A Stand-Alone Target and Multilayered Armor System

**DOI:** 10.3390/polym17040534

**Published:** 2025-02-19

**Authors:** Raí Felipe Pereira Junio, Bernardo Soares Avila de Cêa, Douglas Santos Silva, Édio Pereira Lima Júnior, Sergio Neves Monteiro, Lucio Fabio Cassiano Nascimento

**Affiliations:** Military Institute of Engineering—IME, Department of Materials Science, Praça General Tibúrcio, 80, Praia Vermelha, Urca, Rio de Janeiro CEP 22290-270, Brazil; raivsjfelipe@ime.eb.br (R.F.P.J.); bernardo.soares@ime.eb.br (B.S.A.d.C.); edio@ime.eb.br (É.P.L.J.); sergio.neves@ime.eb.br (S.N.M.); lucio@ime.eb.br (L.F.C.N.)

**Keywords:** ballistic behavior, composites, natural fibers, ballistic simulation, sustainable materials

## Abstract

The development of efficient and sustainable armor systems is crucial for protecting bodies and vehicles. In this study, epoxy composites reinforced with natural lignocellulosic fibers (NLFs) from carnauba (*Copernicia prunifera*) were produced with 0, 10, 20, 30, and 40% fiber volume fractions. Their ballistic performance was evaluated by measuring residual velocity and absorbed energy after impact with 7.62 mm ammunition, as well as their application in a multilayer armor system (MAS). Scanning electron microscopy (SEM) was used to analyze fracture regions, and explicit dynamic simulations were performed for comparison with experimental tests. Residual velocity tests indicated a limit velocity (V_L_) between 213 and 233 m/s and absorbed energy (E_abs_) between 221 and 264 J, surpassing values reported for aramid fabric. All formulations showed indentation depths below the National Institute of Justice (NIJ) limit, with the 40% fiber sample achieving the lowest depth (31.2 mm). The simulation results correlated well with the experimental data, providing insight into deformation mechanisms during a level III ballistic event. These findings demonstrate the high potential of carnauba fibers in epoxy-based polymer composites, particularly as an intermediate layer in MAS, offering a sustainable alternative for ballistic protection.

## 1. Introduction

Over the past couple of years, consumers have become increasingly stringent when it comes to new products made from renewable sources. Consumers are switching to eco-friendly products due to green marketing, updated recycling guidelines, social pressure, and changes in perceived value. Military conflicts and wars have been a constant presence in world history without ever ceasing. The enhancement of defensive measures for individuals and belongings in combat and civil unrest scenarios has progressed alongside the evolution of offensive weaponry [1].

FRPCs, standing for fiber-reinforced polymer composites, are lightweight materials that are resistant to corrosion and have great strength. Because the fibers can be aligned to create strength in various directions, they give fabrics exceptional flexibility. These resources provide cost-effective alternatives to traditional materials and often result in immediate or long-term financial advantages during the lifespan of the product. FRPCs are being used more and more as important structural components in various industries such as the aerospace and automotive sectors, pipelines, bridges, wind energy, military engineering, and ballistic applications. Researchers have suggested utilizing numerous environmentally friendly NLFs [2,3,4].

FRPCs have been gaining prominence as sustainable and efficient alternatives in various industrial sectors [5]. In the context of defense, these materials can be applied in the manufacture of ballistic vests, helmets, and lightweight structures for military vehicles, combining mechanical resistance with weight reduction [6,7]. In the aeronautical sector, they are used for internal aircraft panels and secondary structural components, providing lightness, energy efficiency, and lower environmental impact. In the automotive industry, these composites are used in door panels, interior linings, and even structural parts, contributing to reducing the weight of vehicles and, consequently, improving fuel consumption and reducing CO_2_ emissions [8,9]. For the wind-energy sector, they are used in the manufacture of turbine blades, offering a more ecological and sustainable alternative for the generation of renewable energy, while maintaining high durability and performance [10].

The use of carnauba fibers in the composition of composites can bring countless benefits to those directly and indirectly involved in production, highlighting the possibility of social and economic development in less favored regions through the use of labor applied directly to the cultivation and production of these fibers. At this point, the advantages of using these fibers include the sustainable development of regions with fragile economies, because their cultivation and processing enable the employability of human resources where job offers are scarce [11,12,13].

Thus, studies focused on composites reinforced with carnauba fibers have become interesting, being able to contribute not only to advances in military defense technology but also in conventional or structural applications.

The development of epoxy–carnauba composites represents a significant advance in the search for innovative and sustainable materials for various applications, especially in ballistic armor. Carnauba, a natural fiber abundant in Brazil, gives the material desirable characteristics such as low weight, high mechanical resistance, and good impact absorption capacity, making it a promising alternative to conventional materials used in protection systems [11,12].

In ballistic armor, the efficiency of the material is directly related to its ability to absorb and dissipate the energy of the projectile impact, reducing damage and improving user safety. Composites reinforced with carnauba fibers demonstrate good structural performance, with the ability to withstand significant impacts in addition to maintaining stable dimensionality after impact, which is an essential factor for materials used in personal protective equipment and vehicle armor [12].

In addition to the defense sector, these composites have potential for applications in aerospace, automotive, and civil industries, where lightweight, strong and environmentally sustainable materials are highly desirable. The incorporation of carnauba into epoxy matrices not only improves the mechanical properties of the material, but also contributes to reducing environmental impact, promoting the use of renewable resources and decreasing dependence on synthetic materials [13].

Therefore, the study and application of epoxy–carnauba composites are essential for the development of innovative and sustainable solutions, with the potential to transform various industrial sectors, ensuring safety, efficiency, and sustainability in their applications.

Many researchers have utilized numerical modeling techniques, including the finite element method, with software packages such as Ansys^®^, LS-DYNA^®^, and ABAQUS^®^ [14,15]. The primary goal of the method is to develop a simulation model and forecast the ballistic performance of the target materials [16,17]. The performance of the composite, including matrix damage, fiber damage, and interface damage, is affected when the composite is subjected to severe conditions like static or dynamic loading, different temperatures, and pressure, and these can be analyzed with the finite element method. By using the multiscale approach, which involves replicating the performance of a composite across various scales of time and/or length, it becomes possible to assess characteristics ranging from the nanoscale to the macroscale in composite simulation studies [18].

Shaik and Salvi [19] claim that the representation of all aspects in studies of large structures with millions of components, materials, non-linear structures, and joints is unfeasible due to computational limitations. When a detailed model of a specific area is required, a multiscale modeling technique is used, saving time and computational resources.

Some studies have included the assessment of material properties through representative volume element (RVE) models that make it possible to understand the properties that fiber-reinforced composites may present on a microscale [20,21]. The implementation of RVE models is essential when experimental analyses are unfeasible, serving as a temporary solution for problems such as determining the engineering constants of materials [22,23,24].

In the universe of composite materials simulation, two perspectives are available for obtaining properties: microscale and macroscale. Some studies have evaluated material properties through RVE, making it possible to understand on a microscale the properties that fiber-reinforced composites may present [20,21]. The implementation of RVE models is necessary in situations where experimental analyses to survey properties are generally unfeasible; thus, this approach acts as a provisional source for problem-solving via identifying the engineering constants of materials [22,23]. In the macroscale sphere, simulations of composite materials involve the assembly of sheets, which is a real challenge to be overcome. Different studies have been developed to survey these materials’ properties, including numerical simulation and assembly of the materials [25,26].

In the work developed by Cêa et al. [27] the ballistic properties of hybrid composites with an epoxy matrix reinforced with synthetic aramid fibers and natural fibers of fique were investigated experimentally and numerically. The results obtained showed the potential of hybridized synthetic and natural fibers, and the data collected from the computational simulations demonstrated great similarity with those obtained in the experimental tests.

The assembly of composite sheets is a real challenge to be overcome in relation to the simulation of composite materials. Given this arduous task, different studies have been developed to survey the properties and numerical simulation of these materials [1,25,26].

In the scientific scenario, this work is justified by the characterization of a new material intended for application in ballistic armor, contributing to the understanding of the ballistic properties of the epoxy–carnauba composites evaluated. Thus, such points are presented as original characteristics of this study.

The lack of information regarding the properties presented by epoxy–carnauba fiber composites justifies the need to collect information regarding the ballistic properties of these materials. The search for the development of resistant materials that can be used in engineering applications is an extremely important factor for establishing a product that can replace synthetic materials such as aramid fiber (Kevlar^®^ or Twaron^®^) at an equivalent level. Several composites reinforced with NLFs have been widely investigated as an alternative to replace conventional synthetic fibers [7]. The ballistic performance with a 7.62 mm projectile obtained in the present work was much superior to the Kevlar (58 J) reported by Monteiro et al. [28], highlighting the viability of the application of the composite evaluated in the present study.

In view of this, in present study, tests were performed individually on composite plates with carnauba fiber content ranging from 0 to 40 vol% to measure absorbed energy (E_abs_) post-impact and the limit velocity (V_L_) of the materials. The effectiveness of the MAS with an Al_2_O_3_/Nb_2_O_5_ ceramic front layer followed by a composite plate containing 0 to 40 vol% carnauba fibers placed on a panel with 12 Kevlar^®^ sheets mimicking a level IIIA ballistic vest was assessed. The data obtained were statistically analyzed using analysis of variance (ANOVA) and the Tukey test to detect changes in failure mechanisms and behaviors, and to assess the reliability, significance, and consistency of the results. Finally, a simulation was performed for the purpose of comparison with the experimental analysis. This enabled the understanding of the projectile penetration kinematics, thereby generating an understanding of the action of the deformation mechanisms generated after level III ballistic impact.

## 2. Experiments

### 2.1. Materials

#### 2.1.1. Carnauba Fibers (*Copernicia prunifera*)

**Figure 1:**
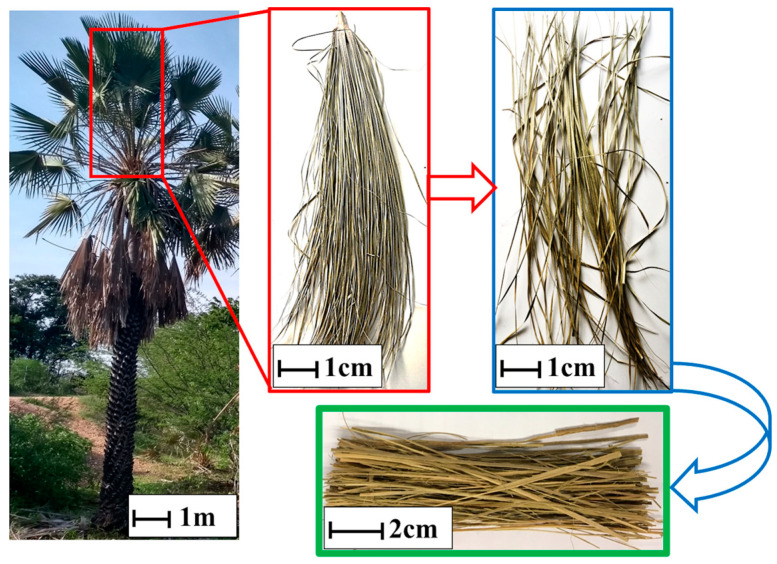
Carnauba leaf fiber and its source tree (*Copernicia prunifera*).

The carnauba fibers used in this work were acquired from rural production and came from the city of Barro, Ceará, Brazil. The material was made available in the form of leaves that were still green. The leaves were immersed in water for 24 h followed by drying in the sun for 12 h and then shredded. The carnauba leaves as received are illustrated in Figure 1 [11].

#### 2.1.2. Epoxy Resin

The material used as the matrix of the composite plate was commercial epoxy resin of the ether type, diglycidyl bisphenol A (DGEBA), hardened with triethylene tetramine (TETA), using a stoichiometric ratio of 13 parts of hardener to 100 parts of resin. It was manufactured by Dow Chemical of Brazil and supplied and distributed by EPOXY FIBER Ltd. (Rio de Janeiro, RJ, Brazil) [12].

#### 2.1.3. Aramid Fabric

The fabric used in this work was supplied by the company LFJ Armoring Trade and Services S.A (Conquext), with S745 weave and 460 g/m^2^ weight, in the form of 8-layer panels glued with chloroprene rubber (NEOKV08) [29].

#### 2.1.4. Ceramic Samples

The first stage of processing the ceramic specimens was the preparation of the powder mixture. Alumina powder (700 g, 94.5% wt.), niobium powder (29.15 g, 3.94% wt.), and liquid PEG binder (11.3 g, 1.53% wt.) were mixed in a ball mill with alumina balls, model MA 500, for 8 h. After the grinding procedure, the mixture was dried in an oven at a temperature of 60 °C for 48 h. Next, the powder was deagglomerated using a mortar and pestle, and it was then sieved through a sieve with an opening of 0.355 mm.

After sieving, the ceramic powder (100 g) was pressed in a hexagonal matrix, formed by two punches and a floating jacket. A load of 12 tons, equivalent to 30 MPa, was applied, as described in the literature, with the aid of an SKAY hydraulic press. The “green” ceramic tablets were sintered in an INTI furnace, model FE 1700, at the IME Ceramic Materials Laboratory. The sintering route used is well established for the preparation of alumina specimens with good densification, and is described below:

- Heating from 25 °C to 158 °C at a rate of 1 °C/min;
- Holding at 158 °C for 1 h;
- Heating from 158 °C to 375 °C at a rate of 1 °C/min;
- Heating from 375 °C to 1000 °C at a rate of 8 °C/min;
- Heating from 1000 °C to 1400 °C at a rate of 5 °C/min;
- Sintering hold at 1400 °C for 3 h, and cooling in the furnace.

The first three stages of this route were responsible for the elimination of the organic binder; therefore, the composition of the material became 96% alumina and 4% niobium. The material presented an average densification of 88.1% on sintering, and an average sintered density of 3.53 g/cm^3^. After sintering, the samples were used to manufacture the MAS intended for ballistic analysis.

### 2.2. Methods

#### 2.2.1. Composite Manufacturing

The composites were prepared with 0, 10, 20, 30, and 40% volume of carnauba fibers. The fibers were subjected to drying in an oven at 30° for 24 h, aiming at a better adhesion of the matrix to the fibers. The compression method was adopted for the manufacture of composite plates. During the manufacture of the composite plates, a metal matrix with an internal volume of 214.2 cm^3^ (15 × 12 × 1.19 cm) and a uniaxial hydraulic press were used.

**Figure 2:**
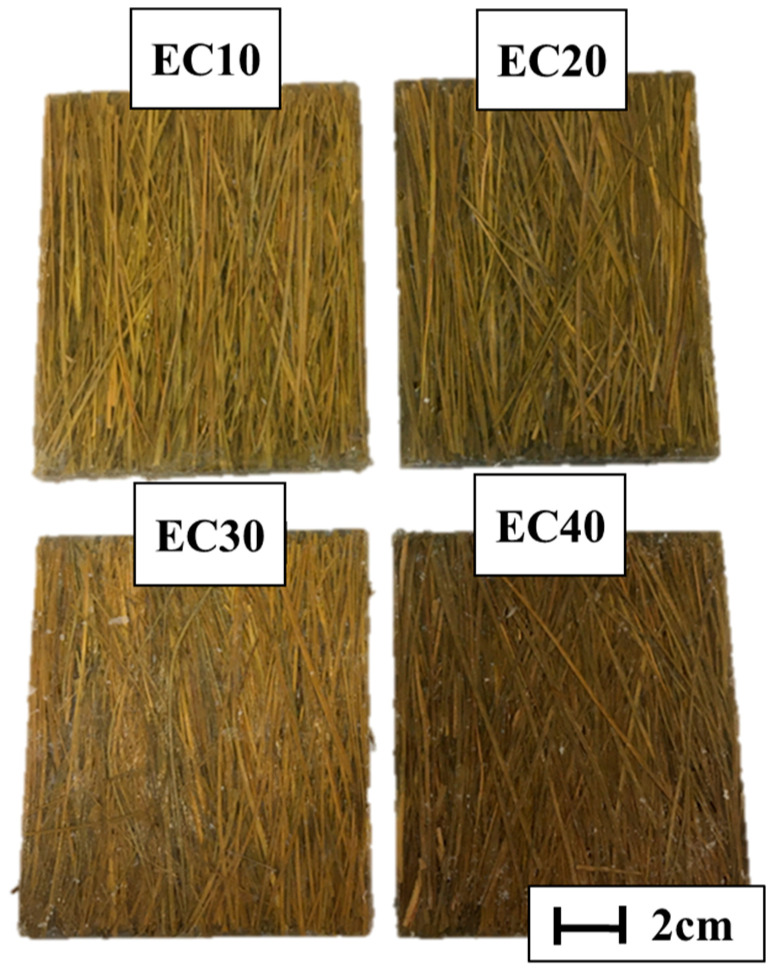
Epoxy–carnauba composite plates with different reinforcement fiber volume fractions.

The set was subjected to a load of 5 tons for 8 h, with the aim of achieving the best material properties as well as reducing the porosity in the composite. The plates that were produced received nomenclatures as shown in Table 1, and the composite plates produced are represented in Figure 2.

#### 2.2.2. Back-Face Signature (BFS) Tests

**Figure 3:**
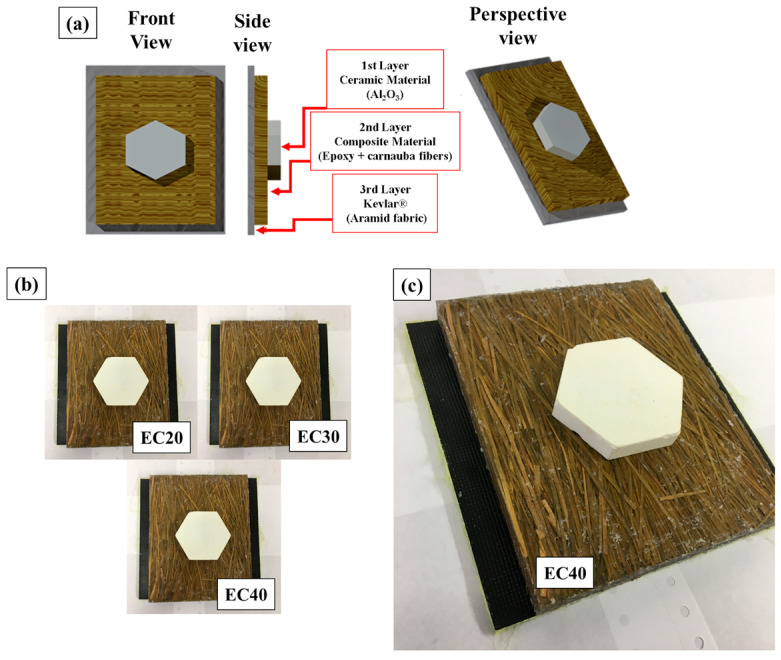
(**a**) Proposed MAS model, (**b**) MAS with epoxy–carnauba composite as secondary layer, and (**c**) MAS with secondary layer of epoxy composite reinforced with 40% carnauba leaf fibers.

The test specimens for the multilayer ballistic test were assembled by gluing the constituent parts together (ceramic tile; composite; aramid fabric) with fast-curing polyurethane (PU) adhesive. The proposed model for multilayer shielding is represented in Figure 3a and the sample obtained in Figure 3b,c.

The EPO and EC10 samples were not evaluated in the trauma tests, as they presented brittle fracture properties. Thus, they presented total fragmentation during the tests, making it impossible to measure the trauma generated after ballistic impact, and they were discarded from the testing.

#### 2.2.3. Scanning Electron Microscopy (SEM)

Scanning electron microscopy was used to characterize the fracture regions of the samples after ballistic impact, with the aim of identifying the main mechanisms of failure and absorption of energy from external forces. For SEM characterization, the samples were coated with gold using V-vacuumable equipment under vacuum for 4 min. The regions of interest were analyzed with a Quanta FEG 250 FEI electron microscope with an acceleration voltage of 5 to 20 kV and spot size of 5.0, with a secondary electron detector.

#### 2.2.4. Residual Velocity Ballistic Test (7.62 × 51 Caliber)

The ballistic test to determine residual velocity aimed to identify the kinetic energy of the projectile and the target’s ability to absorb this energy. For this test, composite plates measuring 15 × 12 × 1 cm^3^ were manufactured, with different volumetric fractions of carnauba fiber reinforcement (0, 10, 20, 30 and 40%). Two plates were produced in each condition, with each plate divided into four parts, thus comprising a total of 8 samples for each condition to be analyzed.

A WEIBEL SL-520P Doppler radar belonging to the Army Evaluation Center (CAEx) was used in the test. Using the data obtained by the radar, the calculation was performed using Equations (1) and (2); 8 shots were considered in each group to determine the residual velocity:

(1) $$E_{abs}=\frac{M(V_{0}^{2}-V_{R}^{2})}{2}$$

(2) $$V_{L}=\sqrt{\frac{2E_{abs}}{M}}$$

where the following apply:

*V*_0_: Projectile velocity immediately before impact;

*V_R_*: Residual velocity of the projectile after piercing the target;

*M*: Projectile mass.

**Figure 4:**
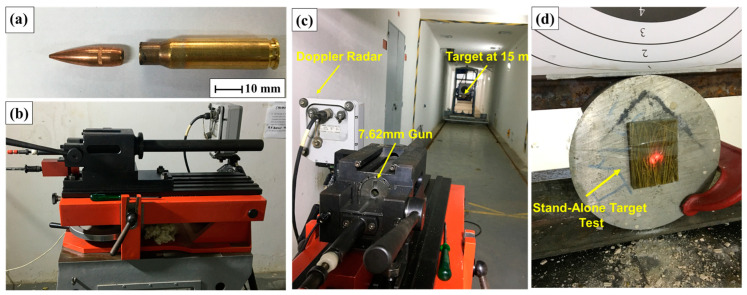
Residual velocity ballistic test setup. (**a**) 7.62 × 51 mm projectile, (**b**) 7.62 mm weapon, (**c**) setup test, and (**d**) epoxy–carnauba composite target.

The test was carried out in a 300-m-long underground ballistic tunnel using commercial 7.62 × 51 mm caliber ammunition, which was fired from a test piece of the same caliber. The targets were positioned 15 m from the tip of the specimen, with an incidence angle of 90°, based on the criteria of regulatory standard NIJ 010106 (2008) [30]. The ammunition used, test specimen, and test setup are shown in Figure 4.

#### 2.2.5. Analysis of Variance (ANOVA)

Statistical tests are essential tools for making data-driven decisions. Among these tests, ANOVA and Tukey’s test are widely used to compare multiple groups. ANOVA was applied as a statistical treatment to assess the ballistic properties of the tested materials. The significance level (*α*) represents the probability of making a type I error, that is, rejecting the null hypothesis (H_0_) when it is true. In the context of an ANOVA test and Tukey’s test, a common significance level is 5% (*α* = 0.05). This means that there is a 5% chance of concluding that there is a significant difference between the groups when, in reality, there is not. The reliability of the test, also called the confidence level, is calculated as 1 − *α*. For a significance level of 5%, the confidence level is 95%. This means that if we were to repeat the experiment several times, we would expect to obtain the same results in 95% of cases. This test used a significance level of 5% or 95% reliability, thus making it possible to compare the influence of the percentage of fibers added to the composite on the properties obtained.

A significance level of 5% (*α* = 0.05) is a standard criterion for rejecting the null hypothesis in statistical tests, and the 95% confidence level indicates that we have high confidence in the results obtained.

The Tukey test was used for pairwise comparison of the averages obtained for each of the treatments used (percentage of fibers). Based on the results, it was possible to reject or not the hypothesis of equality between the means compared through the minimum significant difference (*d.m.s.*), according to Equation (3) [31]:

(3) $$d.m.s.=q\;.\;\sqrt{\frac{QMR}{r}}$$

where the following apply:

*q*: the total studentized amplitude (tabulated value, dependent on the degree of freedom, residue and number of treatments);

*QMR*: mean square of the residue;

*r*: number of repetitions of each treatment.

Using this methodology, it was possible to determine quantitatively and qualitatively in a comparative manner the influence of the volumetric fraction of carnauba fibers applied in the production of the composites, concluding which treatment made it possible to obtain better results.

### 2.3. Numerical Modeling

#### 2.3.1. Johnson–Cook Model

The Johnson–Cook (JC) fracture criterion describes the relationship between the fracture stress of a material and the stress state in terms of stress triaxiality; it is the most commonly used criterion, especially in finite element models [32]. The JC model presents a strong theoretical basis, which is commonly used to predict ballistic behavior by capturing the essential fracture characteristics of ductile metals [33].

The JC model takes the temperature dependence and strain rate sensitivity as a basis governing the quasi-static flow behavior in work hardening of ductile metals [32,33]. Equation (4) illustrates the Johnson–Cook model:

(4) $$\sigma =\left(\sigma_{y}+K\epsilon_{p}^{n}\right)\left\{1-{\left[\frac{T-T_{0}}{T_{m}-T_{0}\;}\right]}^{m}\right\}\left\{1+Cln\left[\frac{\left(\frac{d\epsilon_{p}}{dt}\right)}{{\left(\frac{d\epsilon_{p}}{dt}\right)}_{0}}\right]\right\}$$

where the following apply:

$\sigma_{y}$: Yield stress;

*K*: Hardening coefficient;

*n*: Work hardening exponent;

$T_{m}$: Melting temperature;

$T_{0}$: Reference temperature (ambient);

*m*: Temperature coefficient;

$\left(\frac{d\epsilon_{p}}{dt}\right)$: Plastic deformation rate;

${\left(\frac{d\epsilon_{p}}{dt}\right)}_{0}$: Reference (quasi-static) strain rate;

*C*: Sensitivity parameter.

The JC model was applied to the steel and brass that comprised the projectile geometry. This model was used to describe the plasticity and fracture of both materials [34,35,36]. The parameters used in the model were those presented by other authors in accurate and reliable evaluations; the values obtained for steel and brass are presented in Table 2.

#### 2.3.2. Steinberg–Guinan Model

This is a model widely used for simulations involving resistance, especially for metals subjected to considerable deformation rates. The Steinberg–Guinan (SG) model takes into account the increase in pressure in relation to the increase in yield stress and shear modulus of a given material, or the decrease in these properties with increasing temperature [40]. The yield stress and shear modulus are defined by Equations (5) and (6), respectively:

(5) σ=σ0 1+ σ′pσ0 Pη13+G′TG0T−3001+βϵn

(6) G=G0 1+ G′pG0 Pη13+G′TG0T−3001+βϵn

where the following apply:

*σ*: Yield stress;

$\sigma_{0}$: Initial yield stress;

${\sigma^{\prime }}_{p}$: Derivative of yield stress with respect to pressure;

*P*: Pressure;

*η*: It is the compression defined by the ratio of the initial specific volume and the specific volume;

${G^{\prime }}_{T}$ and ${G^{\prime }}_{p}$: Derivatives;

*G*: Shear modulus;

$G_{0}$: Initial shear modulus;

*T*: Temperature;

*β*: Hardening coefficient;

ϵ: Plastic deformation;

*n*: Hardening exponent.

For the validation of the deformations suffered by the lead that was a constituent material of the projectile, the SG model was applied according to parameters obtained in other investigations. The parameters used to feed the model are represented in Table 3.

#### 2.3.3. Shock Equation of State

To obtain the Hugoniot pressure and energy, the shock state equation relates the shock velocity of the material and the velocity of the displaced particle in the reference material. This relationship is expressed by Equation (7) [41]:

(7) $$U_{S}=C_{0}+S_{1}U_{P}$$

The Mie–Gruneisen EOS parameters obtained in other studies were applied to the projectile materials. The materials were treated as three-dimensionally Lagrangian. The parameters used to simulate the high-velocity impact are represented in Table 4.

#### 2.3.4. Composite Damage Model

The damage model applied to the composite material was based on deformations generated by traction or compression of a given flat surface (intralaminar damage) and stresses for nonlinear shear damage [42]. The evolution of tensile and compressive damage is defined by a simple bilinear relationship based on Equation (8):

(8) $$∆d_{i}=\frac{\varepsilon_{max,i}}{\varepsilon_{max,i}-\varepsilon_{0,i}\;}\;\left[\frac{\varepsilon_{0,i}}{\varepsilon_{ii}^{2}}\right]∆\varepsilon_{ii}$$

The initial strain ($\varepsilon_{0,i}$) and propagation strain ($\varepsilon_{max,i}$) are determined to avoid a mesh-dependent solution, so that the energy dissipation is constant and independent of the volume; $\varepsilon_{max,i}$ is thus changed at an element level.

In this failure mode, the formulations incorporate a gradual reduction in stiffness; so, as the damage increases, the elastic moduli tend to decrease. The permanent and cumulative plastic deformations contributing to failure in the composite can be defined as an increment by Equations (9) and (10):

(9) $$∆\varepsilon_{pl,i}=\left(\beta_{i}-1\right)\;\frac{\sigma_{ii}}{E_{pl,i}^{n}{\left(1-d_{i}\right)}^{2}}∆d_{i}$$

(10) $$\varepsilon_{pl,i}^{n+1}=\varepsilon_{pl,i}^{n}+∆\varepsilon_{pl,i}^{n+1}$$

An erosion model was assigned as a numerical method to automatically remove elements with excessive distortion. This model aimed to ensure the stability of the time steps in the simulation within an acceptable range, promoting the resolution of the simulation. This model was based on the geometric deformation of the element ($\varepsilon_{eff}$) under stress, and the data were obtained from the global deformation components according to Equation (11) [43]:

(11) $$IGS=\varepsilon_{eff}=\frac{2}{3}\;\sqrt{\left(\varepsilon_{exx}^{2}+\varepsilon_{eyy}^{2}{+\varepsilon }_{ezz}^{2}\right)-\left(\varepsilon_{exx}\varepsilon_{eyy}+\varepsilon_{eyy}\varepsilon_{ezz}+\varepsilon_{ezz}\varepsilon_{ezz}\right)+3(\varepsilon_{exy}^{2}+\varepsilon_{eyz}^{2}+\varepsilon_{ezx}^{2})}$$

An instantaneous geometric deformation (IGS) value of 1 was applied for the analysis in question.

#### 2.3.5. RVE Model (Microscale Composite)

To obtain the engineering constants of the composites, numerical simulation was carried out using Ansys Workbench^®^ 2024 R2 software. The modeling of composite materials intended for ballistic simulation was conducted through the use of the Ansys Material Designer^®^ module, with the modeling of the RVE [12,44]. This model addresses the representation of smaller volume elements (microscale) of the material under analysis, accurately presenting the typical properties of the material when used in a macroscale model.

Numerical modeling of RVEs follows a series of modeling relations, according to the geometry specified by the model. Therefore, it was assumed that the composite modeled in the present study presented a unidirectional geometry, with the fibers aligned on a specific axis of the composite [12]. This approach allowed creation of an axis of symmetry with respect to the orientation of the fibers, allowing the following Equations (12)–(15) to be satisfied:

(12) $$E_{y}=E_{z}$$

(13) $$v_{xy}=v_{xz}$$

(14) $$G_{xy}=G_{xz}$$

(15) $$\;G_{xz}=\frac{E_{y}}{2\;(1+v_{yz})}$$

Given these conditions, the engineering constants of the material were obtained by solving 6 loading conditions (3 tensile and 3 shear tests), with a known macroscopic deformation of 0.001. The stiffness matrix presented in Equation 16 of an orthotropic linear elastic material was obtained through the reactions in the boundary conditions applied in the RVE [45]:

(16) $$[D]=\;\left[\begin{array}{c} \sigma_{x}\\ \sigma_{y}\\ \sigma_{z}\\ \gamma_{xy}\\ \gamma_{yz}\\ \gamma_{xz} \end{array}\right]=\left[\begin{array}{ccc} D_{11} & D_{21} & D_{31}\\ D_{12} & D_{22} & D_{32}\\ \begin{array}{c} D_{13}\\ 0\\ \begin{array}{c} 0\\ 0 \end{array} \end{array} & \begin{array}{c} D_{23}\\ 0\\ \begin{array}{c} 0\\ 0 \end{array} \end{array} & \begin{array}{c} D_{33}\;\\ 0\\ \begin{array}{c} 0\\ 0 \end{array} \end{array} \end{array}\begin{array}{ccc} 0 & 0 & 0\\ 0 & 0 & 0\\ \begin{array}{c} 0\\ D_{44}\\ \begin{array}{c} 0\\ 0 \end{array} \end{array} & \begin{array}{c} 0\\ 0\\ \begin{array}{c} D_{55}\\ 0 \end{array} \end{array} & \begin{array}{c} 0\;\\ 0\\ \begin{array}{c} 0\\ D_{66} \end{array} \end{array} \end{array}\right].\;\left[\begin{array}{c} \varepsilon_{x}\\ \varepsilon_{y}\\ \varepsilon_{z}\\ \tau_{xy}\\ \tau_{yz}\\ \tau_{xz} \end{array}\right]$$

Taking as a basis a tensile test in the X direction, a value of $\varepsilon_{x}=0.001$ is applied, while the other deformations are kept at zero, allowing estimation of the first column of the stiffness matrix, according to Equation (17):

(17) $$\left[\begin{array}{c} D_{11}\\ D_{12}\\ D_{13}\\ 0\\ 0\\ 0 \end{array}\right]=\frac{1}{0.001}\left[\begin{array}{c} \sigma_{x}\\ \sigma_{y}\\ \sigma_{z}\\ \gamma_{xy}\\ \gamma_{yz}\\ \gamma_{xz} \end{array}\right]\;$$

This relation is valid for the RVE geometry that occupies [0,Lx] × [0,L]. After resolution, the force in the X direction is integrated on the face located at the coordinate X = LX and subsequently normalized in the area of the face obtaining the stress $\sigma_{x}$. The same procedure is repeated for the other directions, completing the stiffness matrix [D]. The matrix [D]^−1^ depicted in Equation (18) is the compliance matrix, which is used to calculate the RVE engineering constants [46,47]:

(18) $${\left[D\right]}^{-1}=\left[C\right]=\left[\begin{array}{cccccc} \frac{1}{E_{x}} & -\frac{v_{xy}}{E_{y}} & -\frac{v_{zx}}{E_{z}} & & & \\ -\frac{v_{xy}}{E_{x}} & \frac{1}{E_{y}} & -\frac{v_{zy}}{E_{z}} & & & \\ -\frac{v_{xz}}{E_{x}} & -\frac{v_{yz}}{E_{y}} & \frac{1}{E_{z}}\; & & & \\ & & & \frac{1}{G_{xy}} & & \\ & & & & \frac{1}{G_{yz}} & \\ & & & & & \frac{1}{G_{xz}} \end{array}\right]$$

The properties data of the carnauba fibers and epoxy resin used to feed the Material Designer were obtained from previous studies and are shown in Table 5.

The RVE model was proposed for the unidirectionally aligned (UD) fiber material; each volume fraction of the composite evaluated in the present work was modeled in the RVE model. A standard deviation of 0.1 was applied to each evaluated volume fraction of fibers in the composite; a sampling of 3 points per analyzed condition was determined with 5 possible design points [48]. For the orientation of the fibers inside the RVE, a misorientation angle of 1.5° was adopted, with a perturbation algorithm (high fiber volume fraction) and standardized seed of 15,633. A mesh with a maximum element size of 600 µm was generated, with adapted edges and adaptive mesh activated.

#### 2.3.6. Composite Discretization Model (Macroscale)

The macroscale composite model was discretized using the Ansys Composites Pre-Post^®^ (ACP) module in the Ansys Workbench^®^ platform. The representative properties obtained in the RVE model were applied to the composite constituent sheets.

The geometry of the modeled composite plate has the same dimensions as the experimentally tested plates. The model was designed with 50 layers of composite sheets measuring 150 × 120 mm in area by 0.2 mm in thickness.

The composite plates were discretized with 256.000 elements and 263.250 nodes, using Lagrangian meshes [2]. A mesh refinement method was applied to the edges of the layers, with a greater number of elements clustering in the center of the plate close to the ballistic impact region, ensuring the reliability of the model and the properties of the material used.

**Figure 5:**
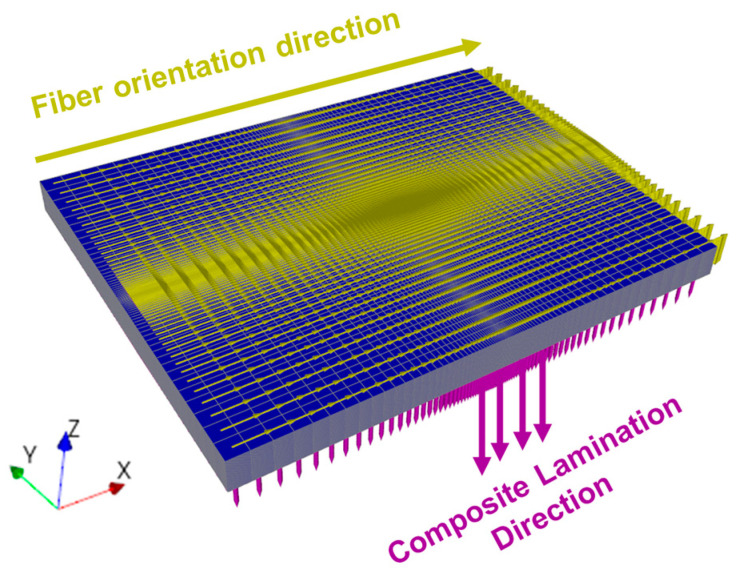
Proposed model for epoxy–carnauba composite laminate.

80 divisions were applied to the edges in the X direction and 64 divisions in the Y direction, both with a division factor of 20. The discretization of the plate can be seen in Figure 5.

#### 2.3.7. Projectile Discretization Model

**Figure 6:**
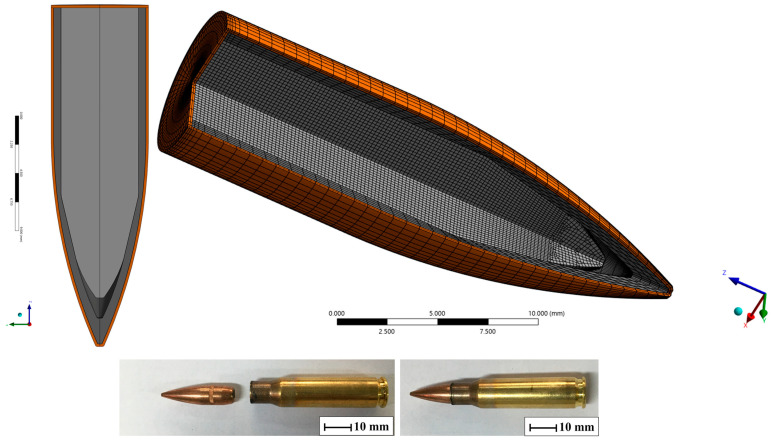
Discretization of the projectile and 7.62 × 51 mm ammunition used.

The projectile was designed using Ansys SpaceClaim^®^, considering all the materials in its composition, including the steel core, lead tip, and brass jacket, with dimensions compatible with previous studies [35,49]. To model the problem, the 7.62 mm FMJ projectile was discretized with 183,680 elements and 199.044 nodes with Lagrangian meshes. The discretization of the projectile can be seen in Figure 6.

For the constituent parts of the projectile (steel, lead, and brass), glued beads were applied between the surfaces in contact, simulating a continuous surface between the materials [34].

#### 2.3.8. Simulation of Ballistic Impact on a Single Target (Explicit Dynamics)

For simulation of ballistic impact on the composites, the nonlinear dynamic analysis module Ansys Explicit Dynamics^®^ from the Ansys Workbench^®^ platform was used. Target plates discretized with the properties of the epoxy–carnauba composite at fractions of 10%, 20%, 30%, and 40% of carnauba fiber reinforcement were observed.

**Figure 7:**
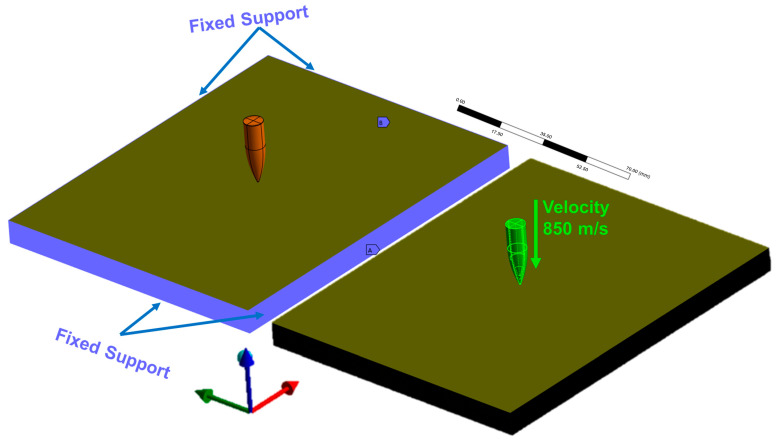
Boundary conditions used in ballistic simulation.

To simulate a level III ballistic impact (7.62 mm), the projectile was positioned 0.5 mm from the target surface with an initial velocity of 850 m/s as per the regulatory standard (NIJ) [30]. Fixed supports were applied to the sides of the target, seeking to avoid displacements and translations after ballistic impact, exempting the target from any effects that could hinder the simulation during impact. The boundary conditions applied in the explicit simulation are represented in Figure 7.

A final simulation time of 1.1 × 10^−4^ s was used, with a time-step safety factor of 0.9, mass scaling activated, and geometric strain limits of 1.

## 3. Results and Discussion

### 3.1. Ballistics Testing of Residual Velocity (Stand-Alone Target)

To verify the resistance of the composite plates produced at each reinforcement volume fraction, a residual velocity test was conducted. The results enabled the estimation of essential data regarding the ballistic properties of composites reinforced with carnauba fibers. Consequently, the absorbed energy (E_abs_) and the limit velocity (V_L_) of each tested sample were determined.

**Figure 8:**
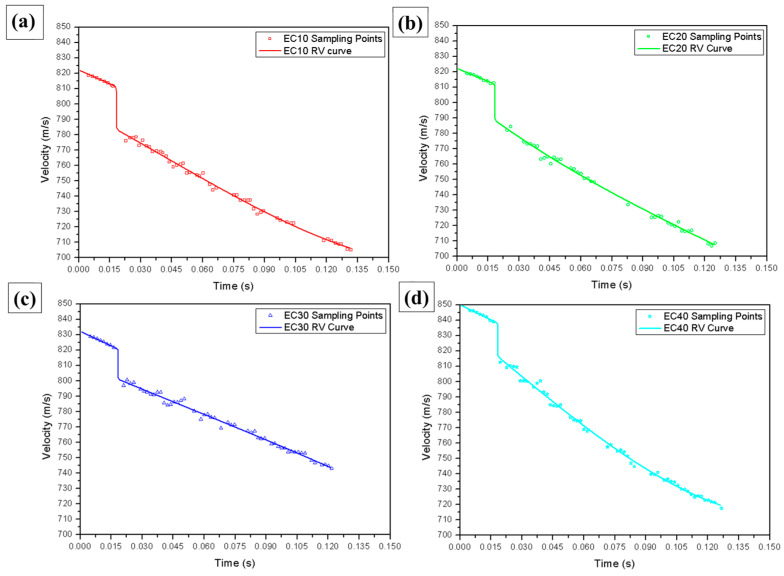
Results of velocities obtained in the ballistics testing of the composites: (**a**) EC10, (**b**) EC20, (**c**) EC30 and (**d**) EC40.

Figure 8 shows the experimental points obtained during the ballistics tests for the tested composite samples. The data were derived from the Doppler radar spectrum, along with the continuous polynomial curve adjusted for the analyzed conditions.

A sudden drop in the projectile’s velocity was observed at around 839 m/s, marking the moment of impact on the target. This velocity was defined as the impact velocity (V_i_), while the minimum velocity reached after impact was the residual velocity (V_r_).

Similar graphs were obtained for the other study conditions. Based on the data from these curves, the limit velocity (V_L_) and absorbed energy (E_abs_) were determined. Table 6 presents the results for the average projectile impact velocity (V_i_), average residual velocity (V_r_), E_abs_ and its percentage relative to the total energy (%E_abs_), and the estimated V_L_.

**Figure 9:**
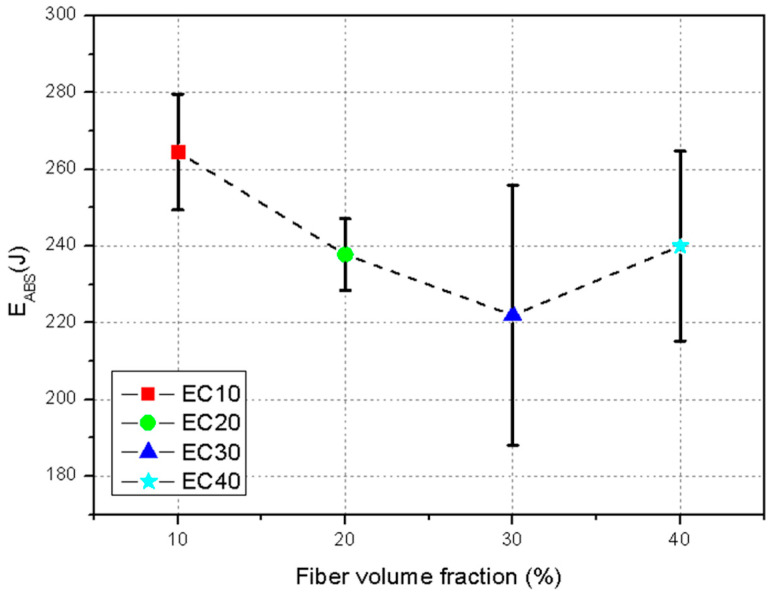
Energies absorbed by the evaluated samples.

A more effective way to evaluate the absorbed energy values of the composites presented in Table 6 is through graphical representation of the test data, as shown in Figure 9.

Table 6 presents the ballistics data obtained for different materials, including pure epoxy (EPO), Kevlar^®^, and epoxy composites reinforced with carnauba fibers in volume fractions of 10% (EC10), 20% (EC20), 30% (EC30), and 40% (EC40). The parameters evaluated include the initial velocity (V_i_), residual velocity (V_r_), absorbed energy (E_abs_), percentage of absorbed energy (%E_abs_), and the limiting velocity (V_L_). These results were used to evaluate the efficiency and the potential use of the tested materials.

Pure epoxy (EPO) exhibited a high residual velocity (V_r_ = 827.0 ± 6.0 m/s) and absorbed relatively low energy (E_abs_ = 241.0 ± 42.0 J), indicating limited resistance to ballistics. The limit velocity (V_L_ = 196.0 ± 32.0 m/s) was lower compared with the composites with carnauba fibers, confirming the low efficiency of pure epoxy for ballistics applications. Kevlar^®^, widely used in armor, showed a residual velocity (V_r_ = 841.0 ± 7.0 m/s) similar to that of pure epoxy, but absorbed significantly less energy (E_abs_ = 58.0 ± 29.0 J). The limit velocity (V_L_ = 109.0 ± 7.0 m/s) was lower than that of all the epoxy–carnauba composites, although the fiber-reinforced materials showed greater capacity to dissipate the projectile’s kinetic energy.

Carnauba fiber-reinforced epoxy composites demonstrated superior performance compared with Kevlar^®^ or pure epoxy. EC10 (10% fibers) showed the highest absorbed energy (E_abs_ = 264.5 ± 15.0 J) and the highest percentage of absorbed energy (%E_abs_ = 8.1 ± 0.55%), reflecting good interaction between the epoxy matrix and the fiber reinforcement at the lowest volumetric fraction. Its limit velocity (V_L_ = 233.4 ± 6.7 m/s) was the highest among all materials tested, demonstrating high resistance to ballistic impact.

With the increase in the fiber volume fraction, there was a reduction in the absorbed energy and in the overall efficiency of the material. EC20 (20% fibers) and EC30 (30% fibers) presented lower absorbed energy values (237.8 ± 9.3 J and 221.9 ± 33.9 J, respectively) and lower percentage efficiency (7.4 ± 4.3% and 6.8 ± 0.9%, respectively). These results may have been associated with interlaminar failures or lower cohesion between the fibers and the epoxy matrix as the reinforcement fraction increased.

The EC40 (40% fibers) demonstrated an interesting balance between energy absorption (E_abs_ = 240.0 ± 24.7 J) and dimensional stability after impact, with a limiting velocity (V_L_ = 222.2 ± 11.2 m/s) close to that of the EC10. Despite presenting a slightly lower percentage efficiency (%E_abs_ = 7.0 ± 0.7%), this composite stood out for its structural resistance and ability to maintain integrity in the face of ballistic impact, which is essential for armor.

In summary, the epoxy–carnauba composites showed greater efficiency than pure epoxy and Kevlar^®^, especially in their ability to absorb kinetic energy. EC40 stood out as the most suitable material, combining good energy absorption and structural stability, reinforcing its potential for use in multilayer armor systems.

Joint analysis of the data from Table 6 and Figure 9, considering the average values, revealed variation in the absorbed energy results. A reduction in the average absorbed energy was observed for composites with 20% and 30% fiber volume, a trend also identified in other studies [52]. In contrast, composites with 40% fiber volume exhibited higher average absorbed energy than those with 10%, 20%, or 30% fiber volume. The composite with 10% fibers showed higher average absorbed energy compared with those with 20% or 30% fiber volume. This behavior may have been associated with the brittle nature of the matrix, leading to formation of fractures on the surface after ballistic impact [53].

To assess the influence of fiber percentage in the composites on absorbed energy, variance analysis (ANOVA) was applied to the obtained values. Table 7 presents the ANOVA results for absorbed energy as a function of fiber percentage in the composites.

Table 7 presents the statistical parameters affecting the reliability of the results. Comparing the calculated F-value (F_calc_ = 3.586) with the tabulated F-value (F_Tab_ = 2.947) shows that F_calc_ < F_Tab_. This confirms with 95% confidence the hypothesis of equality between the treatment means. Consequently, the fiber volume fraction in the composite did not have a direct influence on the absorption of kinetic energy from the projectile.

To determine which composites exhibited variations in absorbed energy concerning the percentage of carnauba fibers, the Tukey test with a 95% confidence level was applied for comparison of means. The minimum significant difference (*m.s.d*) found was 37.0. Table 8 presents the comparison of data between treatment means.

The Tukey test results in Table 8 indicate that the composite with 30% carnauba fibers (EC30—221.9 J) showed a significant difference in its average absorbed energy compared with the composite with 10% fibers (EC10—264.5 J). With a 95% confidence level, this confirmed a significant difference in the absorbed energy values between these two composites as the fiber fraction increased.

**Figure 10:**
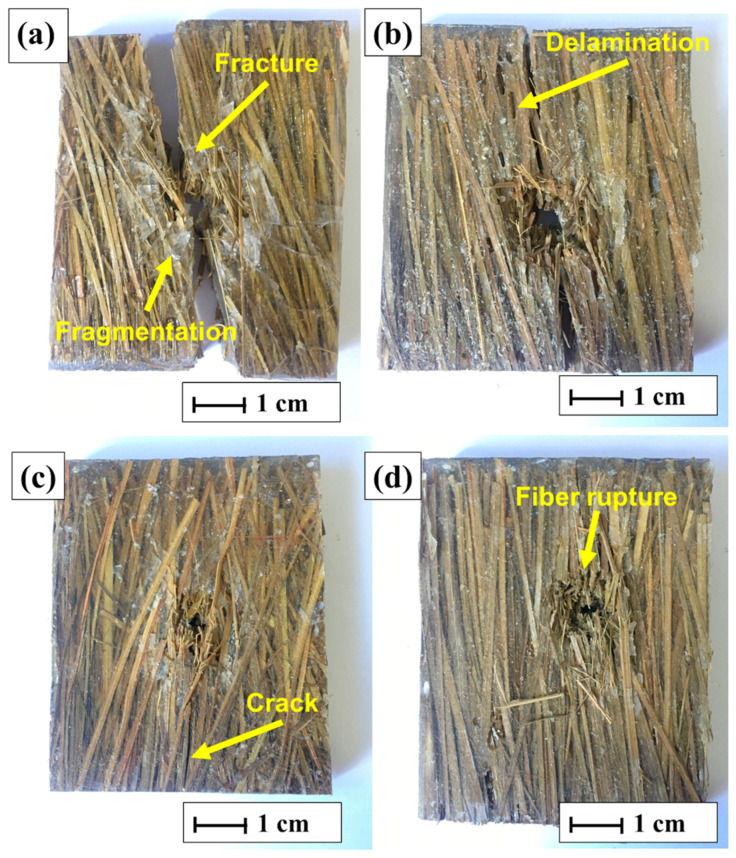
Drilled samples after ballistics test: (**a**) EC10, (**b**) EC20, (**c**) EC30, and (**d**) EC40.

Another important factor when evaluating materials for ballistics shielding is dimensional integrity. In this test, all samples were perforated and penetrated by the projectile, allowing determination of the parameters presented in Table 6. The tested samples are shown in Figure 10.

The plate with 10% carnauba fiber reinforcement exhibited fracture and fragmentation of the brittle epoxy matrix after ballistic impact, as highlighted in Figure 10a. In ballistics applications, fragmentation is an undesirable issue, as it compromises material integrity. Similarly, the plate with 20% fiber volume displayed delamination cracking following the ballistic impact, as shown in Figure 10b.

The samples with 30% fiber reinforcement (Figure 10c) exhibited better dimensional stability than those with 10% or 20% fibers. However, they developed small cracks along their length. These visible cracks suggested potential failures due to delamination, likely to have been caused by exposure to multiple ballistic impacts [54,55].

The 40% fiber-reinforced plates demonstrated better structural integrity after ballistic impact (Figure 10d), with no visible cracks or deformations observed along their surface. This behavior is highly desirable for materials intended for ballistics applications, as supported by several studies [29,56].

The composite with 40% fibers (EC40) stood out as the best among those evaluated, due to its greater dimensional stability after ballistic impact. This can be attributed to the higher proportion of fibers in the polymer matrix, contributing to a more efficient distribution of impact energy. This feature resulted in less permanent deformation and better structural integrity after impact.

Furthermore, the greater fraction of fibers increased the material’s capacity to absorb and redistribute ballistic energy (E_abs_), even though, according to the data presented, there were no significant differences in the absolute values of absorbed energy (E_abs_) between the composites. However, the EC40 structure enabled more optimized behavior, minimizing critical failures and promoting superior performance under impact conditions.

Finally, the synergy between the epoxy matrix and the carnauba fibers in the EC40 composite may have provided a better fiber–matrix interface, ensuring greater use of the mechanical properties of the fibers and better overall performance in ballistic applications.

**Figure 11:**
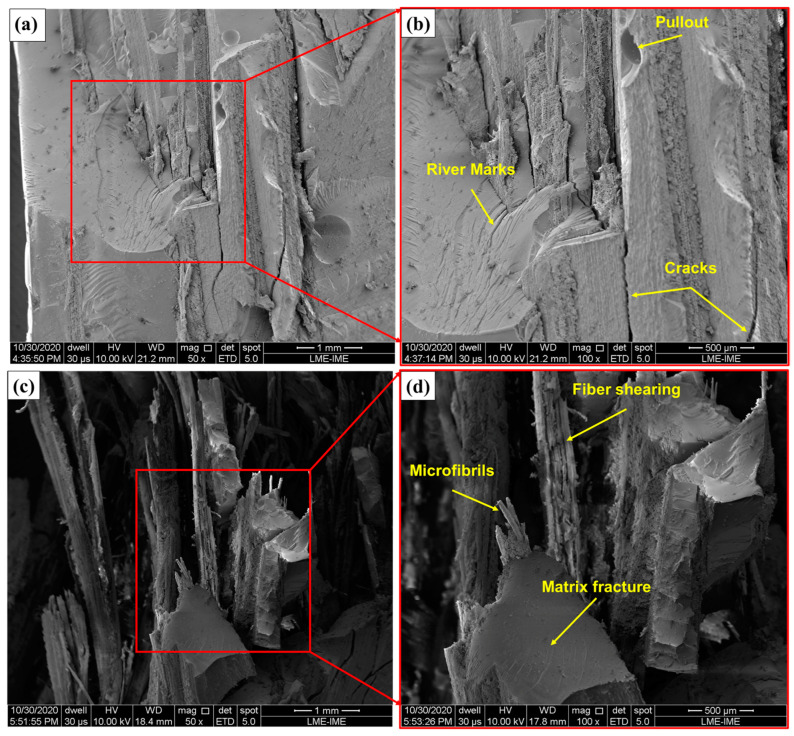
SEM micrographs of the fractured regions of the samples: (**a**) EC10 at 50× magnification, (**b**) EC10 at 100× magnification, (**c**) EC40 EC10 at 50× magnification, and (**d**) EC40 at 100× magnification.

To gain a better understanding of the fracture mechanisms occurring during ballistic impact, SEM micrographs were taken of the regions impacted by the projectile. The images obtained for samples with 10% and 40% fiber volume are shown in Figure 11.

Analyzing the behavior of the composite with 10% fibers (Figure 11a,b), several fracture mechanisms were identified. Although there was no significant difference in the absorbed kinetic energy value, the 10% fiber composite exhibited relatively higher absorption than the other composites. This characteristic was associated with brittle fracture mechanisms of the matrix, combined with absorption of kinetic energy in the reinforcement phase (Figure 11b) [29,57]. However, considering the post-impact appearance, the behavior observed in the composite with 10% fibers was undesirable, indicating that the fiber reinforcement was not effective at this fraction [58].

In the composite with 40% fibers (Figure 11c,d), the ballistic impact region exhibited chaotic behavior, making it difficult to identify the dominant fracture mechanisms due to the higher fiber concentration. As the fiber volume fraction in the composite increased, the impact resistance of the material also improved, leading to the activation of more complex fracture mechanisms [58].

Although the average absorbed kinetic energy of the 40% fiber composites was statistically similar to that of the other composites, these samples exhibited superior dimensional stability after ballistic impact. This characteristic is highly desirable for materials used in ballistics armor.

For all fiber fractions analyzed, the parameters obtained in the residual velocity test exceeded the energy absorbed by Kevlar fabric (58 J) [59]. This, combined with the dimensional stability observed in the 40% fiber composite, underscores the promising performance and quality of the studied composites.

Given the above findings, it can be concluded that when used individually in the dimensions studied, the composite plates reinforced with carnauba fibers were unable to withstand the energy levels generated in the ballistic event analyzed. Therefore, these composites, when applied alone, cannot endure the stresses involved in a level III ballistic event and cannot provide effective protection against 7.62 mm projectiles. For this type of ammunition, the projectile velocity exceeds 800 m/s, as shown in Table 6, whereas the studied materials could only withstand projectile velocities between 213 and 233 m/s.

### 3.2. Back-Face Signature (BFS) Depth Testing of the Multilayered Armor System (MAS)

**Figure 12:**
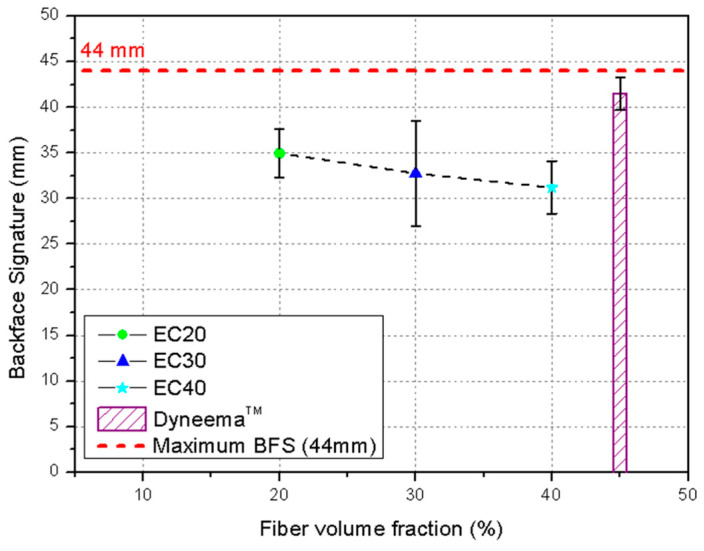
Back-face signature obtained for the evaluated epoxy–carnauba composites and for Dyneema^®^ [29].

The BFS tests aimed to assess the feasibility of using epoxy–carnauba composites as an intermediate layer in an MAS. The trauma results obtained after ballistic impact for the evaluated MAS are presented in Table 9 and Figure 12.

Table 9 presents the ballistic deformation depth (BFS—back-face signature) values for epoxy composites reinforced with carnauba fibers, evaluated as the second layer of a multilayer armor system (MAS) against 7.62 mm ammunition projectiles. The data compare the different volume fractions of fibers in the composites (EC20, EC30 and EC40), while the maximum acceptable deformation limit is established at 44 mm, according to literature reference [29].

The EC20 composites (20% fibers) showed an average deformation depth of 34.9 ± 2.6 mm, well below the acceptable limit of 44 mm. This result indicates good performance in dissipating ballistic impact energy, despite higher deformation than composites with a higher volume fraction of fibers. The EC30 composites (30% fibers) showed an even smaller deformation depth, with an average value of 32.8 ± 5.7 mm. Compared with EC20, this reduction suggests that the increase in fiber fraction improved resistance to impact deformation, which is in line with the expectations for a more structurally reinforced material. The EC40 composites (40% fibers) presented the best results with the lowest deformation depth among the analyzed groups, reaching 31.2 ± 2.9 mm. This performance demonstrated the superiority of composites with a higher volume fraction of fibers in terms of structural stability and their ability to resist the transfer of impact energy to the side opposite the plate.

Pure epoxy (EPO) and composite materials with 10% fibers (EC10) were not evaluated in this configuration, because they are not considered viable or sufficiently resistant for application as a second layer in an MAS.

In general terms, all the composites tested (EC20, EC30 and EC40) presented deformation depths well below the maximum limit of 44 mm established as a safety criterion. This highlights the potential of carnauba fibers as reinforcement in epoxy composites for ballistics armor, with emphasis on the EC40 composite that demonstrated the lowest deformation and consequently, the highest efficiency.

In all the MAS configurations evaluated, the projectile did not penetrate the target. The EC40 samples exhibited the lowest average trauma depth (31.2 mm), while EC20 samples had the highest average depth (34.9 mm). However, considering the sampling deviation highlighted in Figure 12, the samples demonstrated similar behavior. This finding is further confirmed by Table 10, showing the results of ANOVA used to compare the trauma depths resulting from ballistic impact.

**Figure 13:**
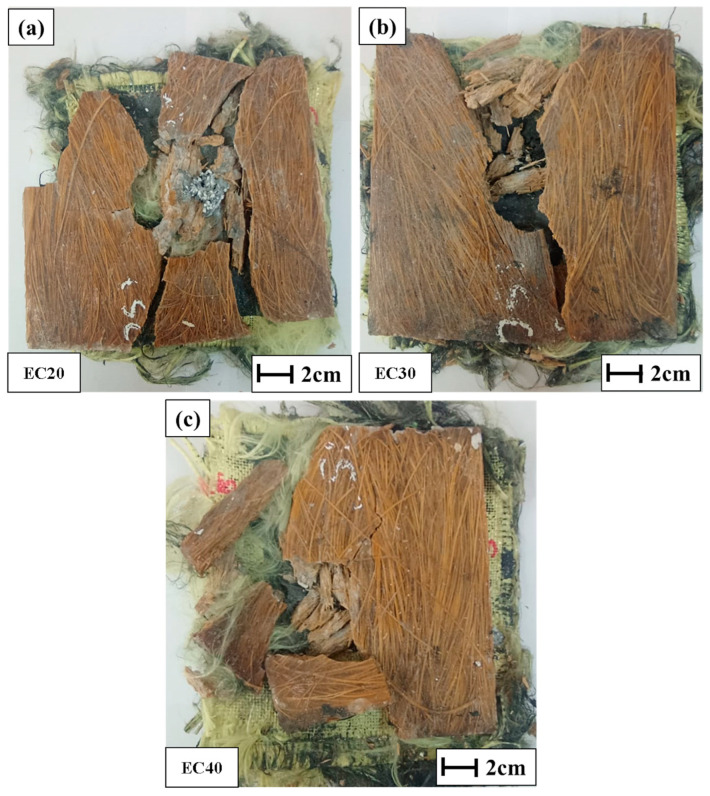
Fractured MAS after 7.62 mm ballistic impact.

Since F_cal_ (1.33) < F_Tab_ (3.68), the hypothesis of a significant difference between the means of the evaluated groups was rejected at a 95% confidence level. However, the EC20, EC30, and EC40 samples showed trauma depths below the maximum limit (44 mm) set by NIJ. The samples after ballistic impact are illustrated in Figure 13.

Comparing the data from this study with the values reported by Da Luz et al. [29] for a Dyneema^®^ (UHMWPE) plate, commonly used in ballistic vests, the epoxy–carnauba composites exhibited lower indentation values. This finding underscores the feasibility of using these materials as a component in an MAS for level III armor.

### 3.3. Computer Simulations

#### 3.3.1. Engineering Constants Obtained with the RVE Model

**Figure 14:**
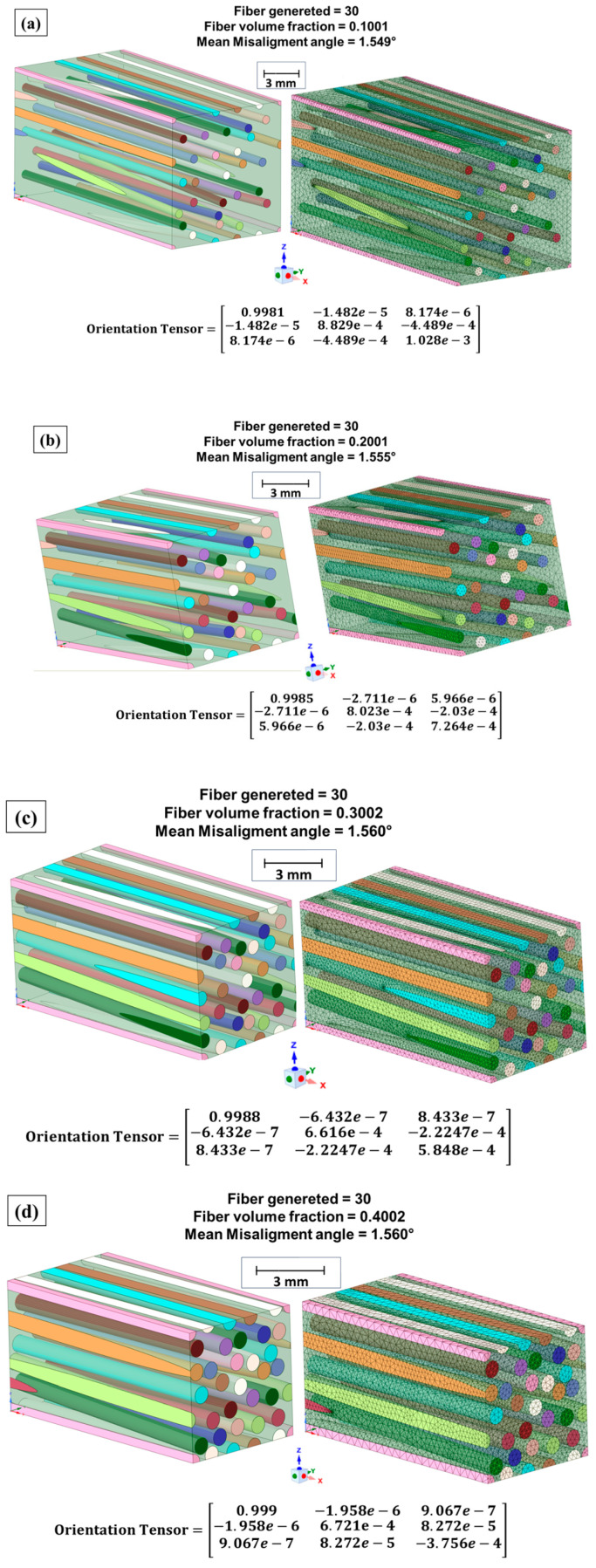
RVE models generated for the epoxy–carnauba composites: (**a**) EC10, (**b**) EC20, (**c**) EC30, and (**d**) EC40.

According to the applied RVE model, representative unit cells of the studied composites were generated, incorporating the mechanical properties of epoxy resin and natural carnauba fiber [12,13]. The obtained representative volumes are illustrated in Figure 14, along with the generated meshes and the tensor orientation of the fibers.

The analysis shown in Figure 14 revealed that the generated elements exhibited a well-distributed arrangement within the RVE, with a regular mesh, ensuring test stability and enhancing the reliability of the obtained properties [13,48]. The engineering constants determined using the RVE model for composites with 10%, 20%, 30%, and 40% carnauba fiber volume are presented in Table 11.

Table 11 presents the engineering constants obtained through the RVE (representative volume element) model for epoxy composites reinforced with carnauba fibers in different fiber volume fractions: 10% (EC10), 20% (EC20), 30% (EC30), and 40% (EC40). The parameters analyzed include the elastic moduli in different directions (E_1_, E_2_, E_3_), the shear moduli (G_12_, G_23_, G_31_), the Poisson’s ratios (ν_12_, ν_13_, ν_23_), and the density of the composites. These values are essential for characterizing the mechanical behavior of the materials.

The elastic moduli (E_1_, E_2_, E_3_) showed a decreasing trend with the increase in the volume fraction of fibers. For the EC10 composite, the moduli had close values (E_1_ = 1648.1 ± 0.88 MPa, E_2_ = 1647.6 ± 0.93 MPa, E_3_ = 1647.6 ± 0.93 MPa), indicating isotropic behavior in the principal directions. At EC40, the values dropped to E_1_ = 1612.4 ± 0.84 MPa, E_2_ = 1610.9 ± 0.87 MPa, E_3_ = 1610.9 ± 0.84 MPa, suggesting that increasing the fiber fraction reduced the material stiffness, possibly due to the less efficient matrix–fiber interface at high concentrations of reinforcement.

The shear moduli (G_12_, G_23_, G_31_) followed the same trend of reduction with the increase in the fiber fraction. At EC10, the values were around 611.59 ± 0.23 MPa, while at EC40, they fell to 602.02 ± 0.22 MPa, evidencing lower shear strength as more fibers were introduced into the composite.

Poisson’s ratios (ν_12_, ν_13_, ν_23_) varied little between samples and remained around 0.34, indicating that lateral deformation was relatively constant in relation to longitudinal deformation, regardless of the fiber volume fraction. This stability in Poisson’s ratios suggests that the elastic behavior of the composite was not significantly affected by the changes in fiber content.

The density of the composites increased with the volume fraction of fibers, going from 1.10 ± 2.23 g/cm^3^ in EC10 to 1.11 ± 2.10 g/cm^3^ in EC40. This increase was associated with the higher concentration of fibers, which had a higher density than the epoxy matrix. This feature is important in applications where weight is a critical factor, such as in the armor industry.

In summary, the table shows that increasing the volume fraction of carnauba fibers reduced the elastic and shear moduli, while increasing the density of the composite. Despite the reduction in stiffness, the values obtained still indicated good mechanical performance, especially for intermediate fiber fractions, as in the case of EC20 and EC30, which presented a balance between stiffness and density. This highlights the potential of these composites for applications where weight reduction and good mechanical properties are essential.

When comparing the modulus of elasticity in direction 1 (E_1_) obtained from the RVE model against data from other studies on epoxy–carnauba composites, it was observed that the values in the present research were relatively lower than those reported by Junio [12]. Additionally, E_1_ values tended to decrease as the fiber volume fraction increased, in contrast with the trend reported by Junio [12]. This variation can be attributed to the significant variability in the mechanical properties of natural lignocellulosic fibers (NLFs) within the composite [60,61]. Factors that influence mechanical behavior, such as surface roughness and interfacial tension between materials, were not explicitly accounted for in the RVE model. Consequently, this limited discretization led to differences between the RVE-predicted properties and those obtained in the experimental tests [62].

The properties obtained in this section were assigned as layers in the modeling of the composite plate, considering fiber volume fractions of 10%, 20%, 30%, and 40%. The homogenized properties of the composites are presented in the following section.

#### 3.3.2. Composite Modeling (ACP)

**Figure 15:**
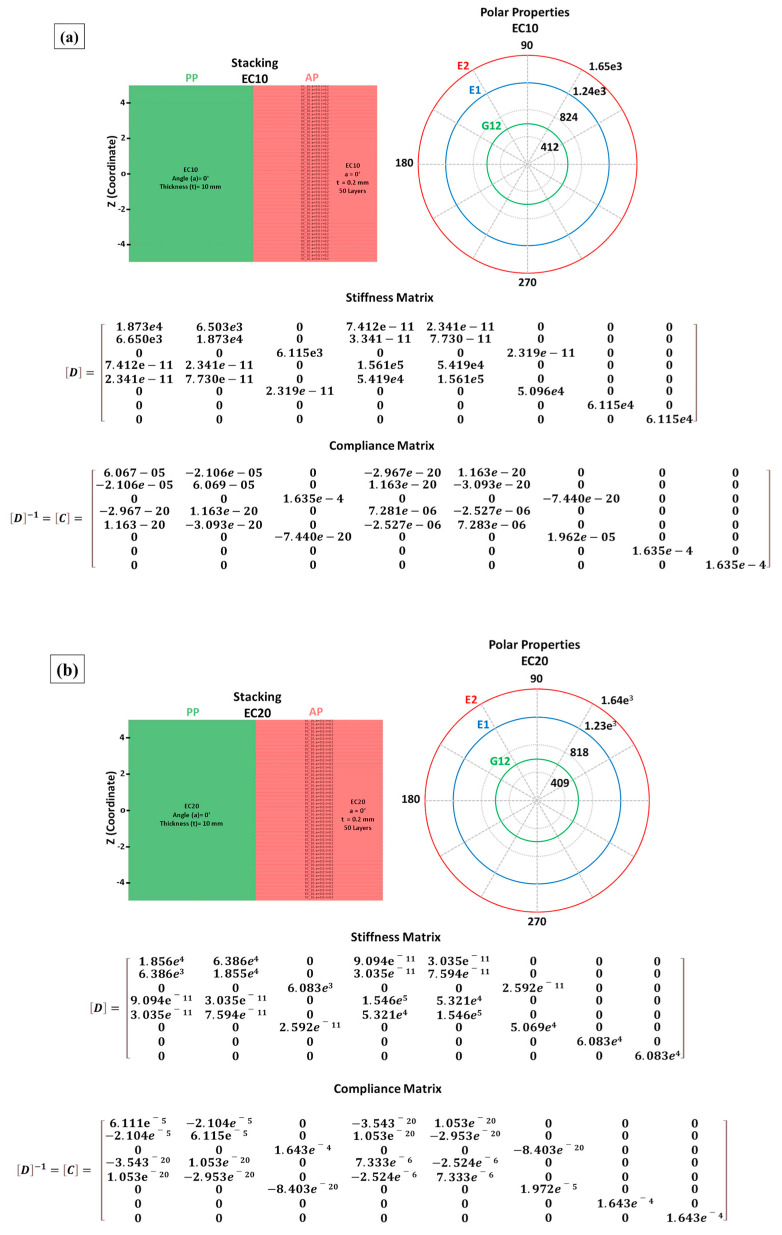

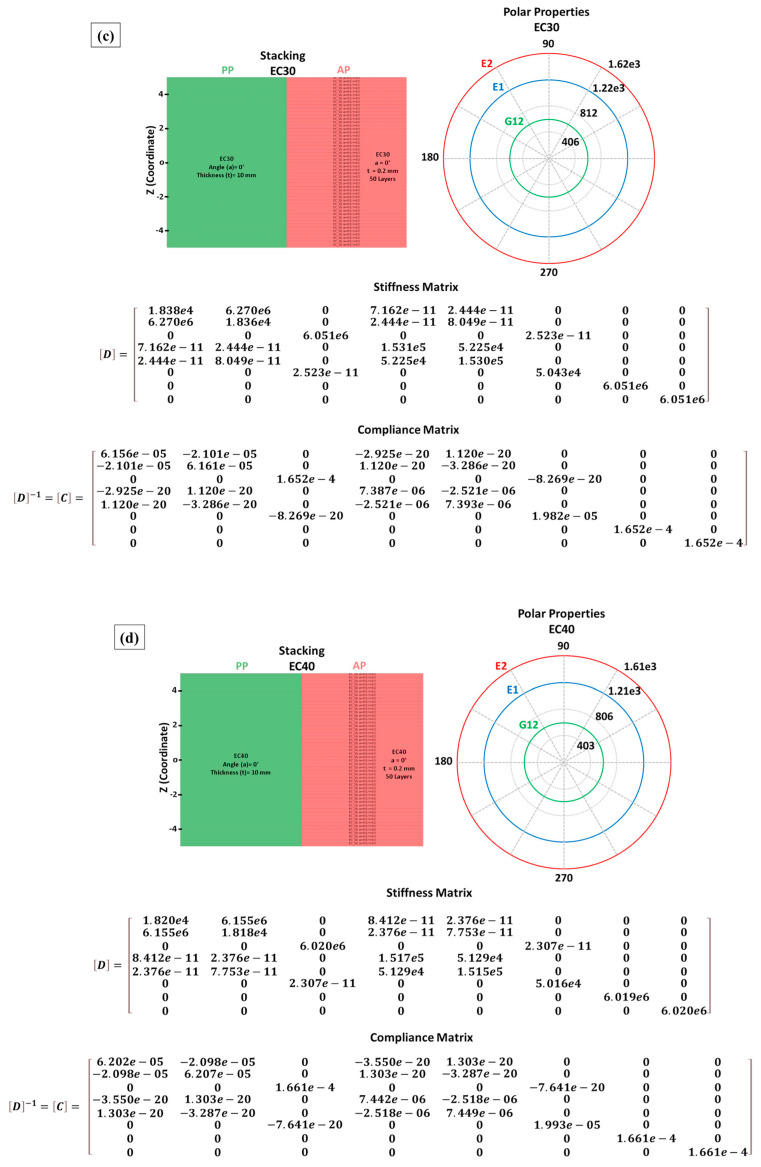
Polar properties, composite assembly, stiffness matrix, and compliance matrix for epoxy–carnauba composites: (**a**) EC10, (**b**) EC20, (**c**) EC30, (**d**) EC40.

The assembly of composites using ACP has proven to be a valuable tool for studying composite laminates, offering various features to predict the mechanical properties of the analyzed materials [25,26]. Figure 15 illustrates the discretized layers, polar properties, stiffness matrix, and compliance for each evaluated composite.

The properties shown in Figure 15 can be better understood by determining the engineering constants related to the laminate [1,63]. This approach was applied, and the obtained values are presented in Table 12.

The data in Table 12 reveal a trend of decreasing laminate properties as the fiber volume fraction increased. This occurred because the model partially applied the rule of mixtures to estimate some properties [64], using the relationships between fiber properties and their fractions as a basis for calculation. Additionally, the laminate exhibited properties similar to those obtained by the RVE model. This similarity arose because the laminate was discretized as unidirectional, as shown in Figure 5, meaning there was no fiber angulation between layers. Consequently, no significant variation in properties occurred between the modeled layers, leading to results consistent with those predicted by the RVE model [1,21].

The composite assembly, along with the mechanical properties obtained for the laminates, was imported into the Ansys Explicit Dynamics^®^ module for ballistics simulation. The data obtained from the Explicit Dynamics simulation are presented in the next section.

#### 3.3.3. Stand-Alone Ballistics Simulation

**Figure 16:**
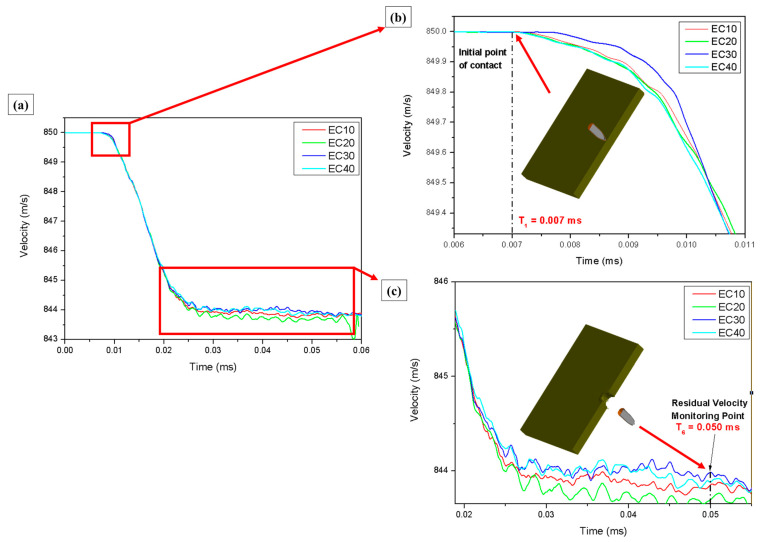
(**a**) Residual velocities obtained in the explicit simulation for the epoxy–carnauba composites. (**b**) Impact velocity collection point and (**c**) residual velocity monitoring point.

According to the NIJ standard, used as a reference to validate the results of this study, the average projectile velocity is 850 m/s. This velocity was adopted as the initial velocity in the simulation [65,66]. The curves obtained from the computer simulation of the ballistics event are shown in Figure 16.

Figure 16 shows that the initial projectile velocity (850 m/s) was observed at T_0_ (0 ms) under all simulation conditions. After T_1_ (0.007 ms), a decrease in projectile velocity began, marking the moment of initial contact between the projectile and the target, as highlighted in Figure 16b. The residual velocity in the simulation was determined based on the velocity recorded at T_6_ (0.050 ms). The residual velocities obtained for each test at T_6_ are presented in Table 13, alongside the average values from the experimental tests for comparison.

As detailed in Table 13, the initial velocity in the experiments was slightly lower than that used in the simulations. The residual velocity in the experiments was considerably lower than in the simulations, implying greater absorption of energy in the real tests. The energy absorbed by the materials in the experiments was much higher than that predicted by the simulation, which can be attributed to simplifications or limitations of the numerical model used.

Among the possible causes for the variation in the properties presented in the numerical model, material properties (elasticity modulus, tensile strength, density, etc.) were generally assumed to be constant and homogeneous, while in the real world, they can vary due to factors such as heterogeneity of the epoxy–carnauba matrix and uneven distribution of fibers. The real behavior of reinforcements such as carnauba fibers includes complex interactions between the matrix and the fibers, which may not be adequately represented in simulation.

During ballistic impact, materials experience complex dynamic behaviors (such as delamination, cracking, and plastic deformation) that can be difficult to model accurately. Also, the simulation may not accurately capture projectile contact and interactions with the material, such as friction and energy dissipation due to local deformation.

Numerical ballistics simulations have advanced significantly, providing greater accuracy in predicting the behavior of projectiles and targets. However, there are still computational limitations that affect the fidelity of analysis compared with experimental tests. Detailed ballistics models require high processing power, especially when involving methods such as the finite element method (FEM).

The need for refined meshes and complex calculations can make simulations slow, limiting their scalability to multiple scenarios. Ballistic materials such as metal alloys, ceramics, and polymers have highly non-linear dynamic behaviors that are difficult to represent accurately. Models that do not correctly include phenomena such as dynamic fatigue, thermomechanical effects, and phase transitions can lead to inaccurate predictions. The fragmentation behavior of the target material and the projectile are still difficult to model with high accuracy, especially when secondary fragmentation and multiple interactions between particles occur. Lack of precision in this aspect can result in discrepancies between simulated and experimental results.

Although ballistics simulations have advanced significantly, there are still computational challenges that impact their accuracy and applicability. The integration of new computational techniques, improvements in materials modeling, and the use of AI can provide more reliable and efficient simulations. However, to ensure the accuracy of the models, rigorous validation with experimental data is essential, maintaining a continuous cycle of improvement between simulation and physical testing. With these improvements, computational models can become increasingly robust tools for applications in defense engineering, armor development, ammunition design, and forensic investigations, reducing costs and optimizing ballistics processes.

The simulation process reached a total time of 0.06 ms. During the first 0.03 ms, the projectile’s velocity decreased exponentially, followed by a steady decline until it came to a complete stop. Comparing the simulation results with the experimental tests, a difference in residual velocity was observed. The experimental values showed a lower residual velocity than those predicted by the simulation model [46,67]. Additionally, the impact velocities in the experimental tests were lower than the velocity used in the simulation, which naturally led to lower residual velocities in the experiments. It is also important to note that the proposed model does not account for external factors such as gravity and air friction. As a result, the simulation did not include reductions in velocity due to these interactions. Furthermore, the model does not consider brittle fracture-induced cracks, which play a significant role in dissipating the kinetic energy of the projectile after impact [29].

Numerical ballistics simulation has become an essential tool to complement and optimize experimental tests in various applications, from the military industry to the development of ballistics protection materials. The correlation between simulations and experimental results allows the validation of computational models and reduces the need for expensive physical tests. Numerical methods such as the finite element method (FEM) are used to predict the behavior of projectiles when impacting different targets. These computational models take into account variables such as initial projectile velocity, impact angle, material composition, and thermodynamic effects. Comparing these results with real experiments, one can adjust the model’s parameters and improve its accuracy. For the development of armor, numerical simulation allows evaluation of different compositions and thicknesses of materials before building real prototypes, while in military and security training, numerical models can help predict the effectiveness of protective barriers and defensive structures in different scenarios. By integrating numerical simulations with experimental data, ballistics research gains in efficiency, cost-effectiveness and reliability, allowing faster and more accurate technological advances in this area.

**Figure 17:**
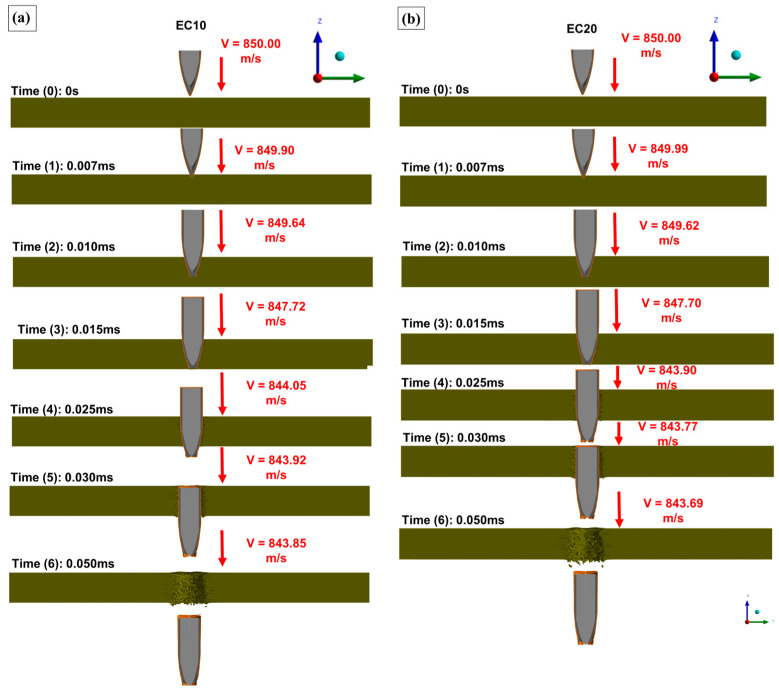

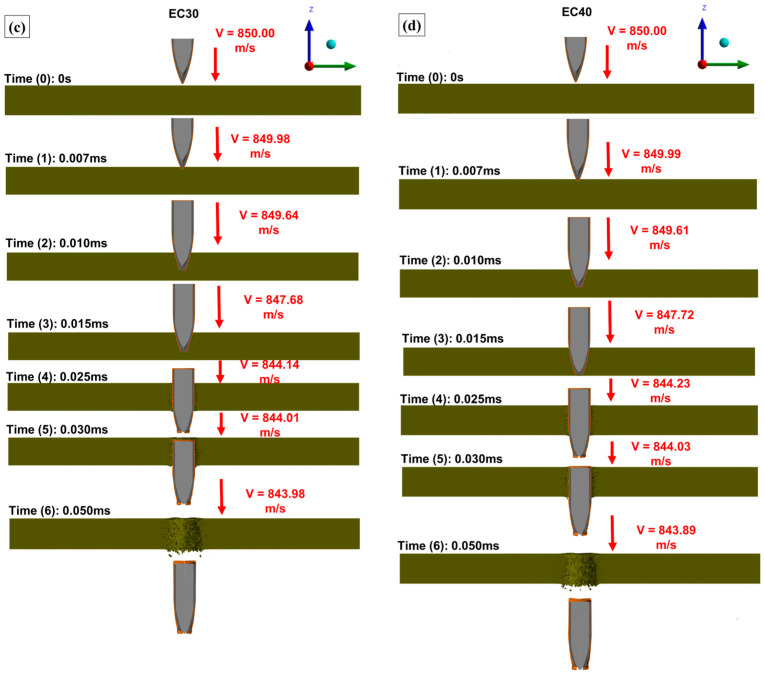
Kinematics of projectile penetration in epoxy–carnauba composite targets: (**a**) EC10, (**b**) EC20, (**c**) EC30, (**d**) EC40.

The proposed model enabled the generation of images depicting the projectile’s kinematics upon impact with the target plate at the specified velocity [42,68]. Figure 17 illustrates the ballistic impact phenomenon observed under the four evaluated conditions, with the projectile–target assembly plotted in a cross-sectional view for analysis.

**Figure 18:**
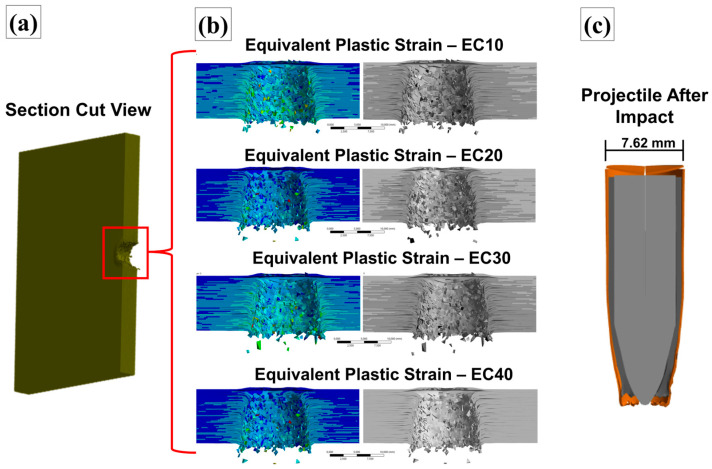
(**a**) Cross-sectional view of the composite plate pierced by the projectile. (**b**) Enlarged view of the cross-section with details of the deformation generated in the composite plates, with volumetric fractions of 10%, 20%, 30%, and 40%, after the passage of the projectile. (**c**) Detail of the 7.62 mm projectile deformed after impact.

In Figure 17, the projectile penetration process in the evaluated targets can be identified. The selected simulation time frames shown in Figure 17a–d represent different stages of projectile penetration, highlighting the precise position of the projectile at each time interval. Additionally, flaws in the modeled layers due to projectile penetration were observed, enabling comparison between the projectile’s perforation of the targets and the residual velocities plotted in Figure 16. This visual representation provided a better understanding of the modeled behavior during ballistic impact [69]. A plot of the damaged sections of the composites was generated, and the equivalent plastic strain (EPS) was monitored using rainbow and grayscale representations. This plot is shown in Figure 18.

Analyzing Figure 18, the failure behavior of the composites was found to be similar to that observed in the experimental tests. The projectile fully perforated all plates during the simulation, mirroring the results obtained in the experimental analyses (Figure 10). The rupture of the layers primarily occurred due to shearing, with plastic deformation regions surrounding the failed areas, highlighted in light blue tones [29,58]. Additionally, grayscale plotting revealed delamination between the modeled layers, characterized by dark longitudinal regions visible in the figures. Fragments from the plates, which emerged after the ballistic impact, were also identified, and are likely to have resulted from layer failures in the modeled composite.

Another important factor to highlight is that the EC10 and EC20 samples exhibited macroscopic longitudinal cracks after ballistic impact. However, this behavior was not replicated in the simulation model, as the EC10 and EC20 simulations did not account for brittle fracture mechanisms due to the absence of brittle fracture modeling. A plastic deformation process was observed in the projectile, as highlighted in Figure 18, resulting from its interaction with the composite layers [66]. Significant degradation was identified in the brass and lead components of the projectile was identified, attributed to the erosion processes applied in the model. In contrast, the steel core of the projectile exhibited minimal deformation compared with the other metals, due to its higher mechanical strength. Overall, both the projectile and the targets demonstrated acceptable plastic deformation behavior, providing valuable insights into the deformation mechanisms of the materials involved in the ballistic event [70].

**Figure 19:**
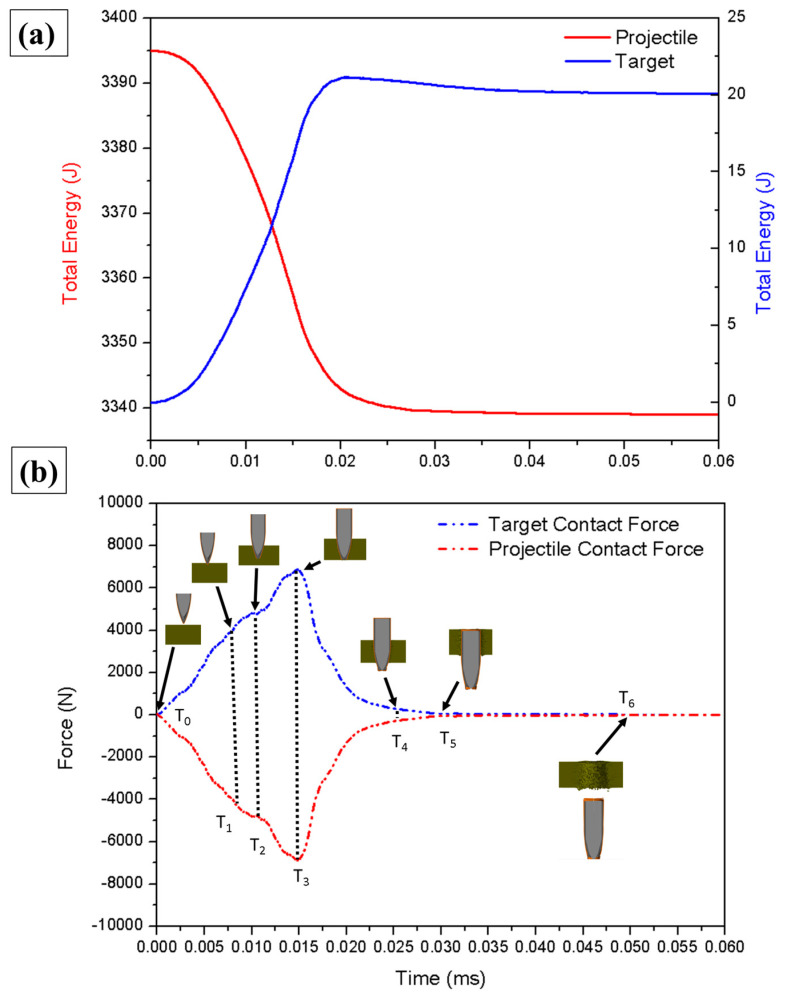
(**a**) Total energy in the target and projectile according to the simulation. (**b**) Graphs of contact forces generated on the target and projectile during the simulation.

Another way to understand the efforts generated during the simulation in question is by plotting the total energy of the event and recorded contact forces, as shown in Figure 19a and Figure 19b, respectively. The energy graphs were plotted for a projectile velocity of 850 m/s, with the projectile mass reported by the simulation as (9.4 × 10^−^^3^ kg), generating a projectile kinetic energy of 3395 J delivered to the target [71]. In the case of the ballistics event in question, the main energy absorption mechanisms were shear, friction between the target and projectile, and stretching or failure due to traction in the fiber region. The compression caused by the projectile on the target also absorbed part of the kinetic energy, and the rest of the energy from the process dissipated via other mechanisms [72,73].

Figure 19b shows the contact forces on the target (C_FT_) and projectile (C_FP_) that occurred during the ballistic event, highlighting the position of the projectile in relation to the simulation times (T_0_, T_1_, T_2_, T_3_, T_4_, T_5_, T_6_). A contact time of 0.043 ms was identified, being the time elapsed from the beginning of contact (T_0_ = 0 ms; C_FT_ = 0 N) of the projectile with the target until exit (T_6_ = 0.050 ms; C_FT_ = 0 N). A sudden increase in the contact force was initially observed until the time interval T_1_ (0.007; C_FT_ = 3617 N), at which time the projectile made contact with the target and promoted the first shear fractures; this increase in contact force represents the target’s resistance to penetration at instant T. At T_2_ (0.010 ms; C_FT_ = 4807 N) there was a variation in the contact force; this characteristic indicates the passage of the sharp tip of the projectile as the thickest section of the projectile (side) comes into contact with the fractured region of the target. At T_4_, the greatest contact force was identified during the simulation process (C_FT_ = 6922 N) at the point representing the largest area of surface contact between the projectile and the interior of the target. This time translated into the fracture of the last layer to be broken from the composite, after which there was a significant reduction in the target contact force [74,75]. At T_4_ (0.025 ms; C_FT_ = 290 N) and T_5_ (0.030 ms; C_FT_ = 49 N), rupture of the last layer of the composite had already occurred and the contact force between the projectile and the target translated into just the friction between the surfaces of materials. This fact highlights the decision taken to monitor this time interval as a residual velocity monitoring point.

## 4. Summary and Conclusions

In this study, for the first time, epoxy composites with varying carnauba leaf fiber volume fractions were tested with autonomous ballistics tests using 7.62 mm caliber ammunition. The use of MAS was examined by conducting BFS tests, and the fracture surfaces were studied using SEM. In addition, explicit computational simulation of ballistic impact was conducted to compare the results. The results led to the following conclusions:

- The limiting velocity and absorbed energy of composites with 40% carnauba fibers by volume showed no significant difference compared with the values reported by other authors. The obtained parameters were approximately 222 m/s and 240 J. For the fractions analyzed in the present work, the parameters obtained for limit velocity (V_L_) (213~233 m/s) and absorbed energy (E_abs_) (221~264 J) were higher than the values established in the literature for the aramid fabric (109 m/s and 58 J). This fact shows the viability of using epoxy–carnauba composites in ballistic applications. Composites with 40% fiber volume showed better dimensional stability after ballistic impact, a desirable characteristic for materials used in ballistics applications;
- Utilizing carnauba leaf fiber-reinforced composites as a second layer in the MAS with varying reinforcement fractions led to no perforation and to BFS depths under 44 mm, meeting the maximum trauma limit set by the NIJ standard. The samples with 40% fiber volume presented a lower mean trauma value than the others (31.2 mm). It is noteworthy that when comparing the values obtained for the composites with 40% volume against a Dyneema^®^ plate, the carnauba epoxy composites presented lower indentation. Additionally, the MAS showed increased physical integrity when using 40 vol% fiber-reinforced composite plates, indicating that this percentage is ideal for MAS usage;
- Fractographic analysis showed different energy absorption mechanisms associated with the carnauba fibers and the epoxy matrix. For EC10, predominant brittle fracture mechanisms were evidenced, such as river marks, longitudinal cracks, and brittle fracture of the matrix, demonstrating the brittle behavior of the composite. For EC40, ductile fracture mechanisms such as fiber shearing, matrix detachment, and pullout began to predominate. This behavior indicated that as the fiber content in the material increased, more complex fracture mechanisms also began to act;
- The application of the RVE method through Ansys Material Designer^®^ was extremely important to obtain the engineering constants of the reinforcement conditions applied experimentally, since there is a lack of such information in the literature. The properties obtained were essential for feeding the simulation processes, making these possible in a relatively short time;
- Modeling using Ansys Composites Pre-Post^®^ (ACP) proved to be a very useful tool for studying composite laminates, making it possible to assemble the materials under study with optimized configurations. This enabled resolution of the simulations in shorter times, resulting in lower computational cost for the simulation;
- Ballistic simulation conducted via Ansys Explicit Dynamics^®^ provided a better understanding of the energy levels, velocity, penetration kinematics, deformation mechanisms, and failures in a level III ballistic event. The residual velocities obtained in the simulation presented lower averages than the experimental data;
- However, the differences between the velocities were at acceptable levels, indicating the possibility of applying the methodology for predicting ballistic behavior to determine the residual velocity of projectiles in level III ballistic impacts;
- In order to increase the scalability of epoxy–carnauba composite production, more advanced composite production techniques such as vacuum-assisted manual lamination or resin infusion can be used; such methods can be investigated in future research, as well as investigating the possibility of producing composites with a volume fraction greater than 40% in carnauba fiber reinforcement, evaluating the mechanical behavior of composites of other polymer matrices reinforced with carnauba fibers, and studying the effect of functionalization with graphene oxide in the assembly of the carnauba fiber matrix.

## Figures and Tables

**Table 1:** Nomenclature used for composites plates.

Nomenclature	Materials	Fiber Volume Fraction
EPO	Epoxy resin	0
EC10	Epoxy resin + Carnauba leaf fibers	10%
EC20		20%
EC30		30%
EC40		40%

**Table 2:** Johnson–Cook parameters for materials modeling of the bullet.

Properties	Materials		Reference
	Jacket Brass	Steel	
Density (kg/m^3^)	8450	7830	[37]
Shear Modulus (MPa)	40,000	81,800	[38,39]
Initial Yield Stress (MPa)	112	792	
Hardening Constant (MPa)	505	510	
Hardening Exponent	0.42	26	
Strain Rate Constant	0.009	0.014	
Thermal Softening Exponent	1.68	1.03	
Melting Temperature (K)	1189	1793	

**Table 3:** SG Parameters for materials modeling of the bullet.

Properties	Material	Reference
	Lead	
Density (kg/m^3^)	11,340	[37]
Shear Modulus (MPa)	8600	
Initial Yield Stress Y (MPa)	8	
Maximum Yield Stress Ymax (MPa)	100	
Hardening Constant B	110	
Hardening Exponent n	0.52	
Derivative dG/dP G’P	1	
Derivative dG/dT G’T (MPa/C)	−9.976	
Derivative dY/dP Y’P	0.0009304	
Melting Temperature (K)	486.85	

**Table 4:** Shock EOS linear parameters for materials modeling of the bullet.

Properties	Materials		Reference
	Jacket Brass	Steel	
Gruneisen Coefficient	2.04	2.74	[37]
Parameter C1 (m/s)	3726	2006	
Parameter S1	1.434	1.429	
Parameter Quadratic S2 (m/s)	0	0	

**Table 5:** Mechanical and physical properties of fiber and matrix.

Properties	Materials		Reference
	Carnauba Fibers	Epoxy	
Density (kg/m^3^)	1130	1100	[12,13]
Young’s Modulus (MPa)	1540	1660	
Poisson’s Ratio	0.32	0.35	
Bulk Modulus (MPa)	1425.9	1844.4	
Shear Modulus (MPa)	583.33	614.81	
Tensile Ultimate Strength (MPa)	69.8	29.3	
Maximum Plastic Strain	0.057	0.012	

**Table 6:** Parameters for stand-alone target test.

Samples	V_i_ (m/s)	V_r_ (m/s)	E_abs_ (J)	% E_abs_	V_L_ (m/s)	Reference
EPO	850.0 ± 2.0	827.0 ± 6.0	241.0 ± 42.0	-	196.0 ± 32.0	[50]
Kevlar^®^	848.0 ± 6.0	841.0 ± 7.0	58.0 ± 29.0	-	109.0 ± 7.0	[51]
EC10	817.07 ± 6.2	782.9 ± 6.4	264.5 ± 15.0	8.1 ± 0.55	233.4 ± 6.7	PW
EC20	812.1 ± 17.3	781.3 ± 16.9	237.8 ± 9.3	7.4 ± 4.3	221.4 ± 4.3	PW
EC30	819.2 ± 9.2	790.8 ± 7.4	221.9 ± 33.9	6.8 ± 0.9	213.4 ± 15.9	PW
EC40	835.6 ± 3.7	805.4 ± 5.7	240 ± 24.7	7.0 ± 0.7	222.2 ± 11.2	PW
PW—Present Work						

**Table 7:** ANOVA of the energy absorbed by the evaluated composites.

CV ^a^	DF ^b^	C	SS ^c^	MS ^d^	F_Calc_^e^	F_Tab_^f^
Treatment	3	1,883,179.4	6915.9	2305.3	3.586	2.947
Residues	28		18,249.7	651.7		
Total	31		25,165.7			

^a^ Cause of variation; ^b^ degrees of freedom; ^c^ sum of squares; ^d^ mean square; ^e^ F—calculated; ^f^ F—tabulated.

**Table 8:** Average values of the Tukey test for energy absorbed by composites.

Samples	EC10	EC20	EC30	EC40
EC10	0	26.7	42.5	24.5
EC20	26.7	0	15.8	2.1
EC30	42.5	15.8	0	18.0
EC40	24.5	2.1	18.0	0

**Table 9:** BFS depth of indentation of epoxy composites reinforced with carnauba leaf fibers tested as a secondary MAS layer against 7.62 mm ammunition.

Samples	BFS (mm)	Reference
EPO	Not evaluated	PW
EC10	Not evaluated	PW
EC20	34.9 ± 2.6	PW
EC30	32.8 ± 5.7	PW
EC40	31.2 ± 2.9	PW
BFS Lethal	44	[29]
PW—Present work		

**Table 10:** ANOVA of the BFS depth from the ballistics tests of MAS with carnauba–epoxy composites as a secondary layer.

CV ^a^	DF ^b^	C	SS ^c^	MS ^d^	F_Calc_ ^e^	F_Tab_ ^f^
Treatment	2	19,575.6	42.9	21.4	1.33	3.682
Residues	15		242.2	16.1		
Total	17		285.2			

^a^ Cause of variation; ^b^ degrees of freedom; ^c^ sum of squares; ^d^ mean square; ^e^ F—calculated; ^f^ F—tabulated.

**Table 11:** Engineering constants obtained via the RVE model for the carnauba–epoxy composites evaluated.

Properties	Samples			
	EC10	EC20	EC30	EC40
Fiber Volume Fraction (%)	0.1 ± 0.01	0.2 ± 0.01	0.3 ± 0.01	0.4 ± 0.01
E_1_ (MPa)	1648.1 ± 0.88	1636.2 ± 0.91	1624.3 ± 0.87	1612.4 ± 0.84
E_2_ (MPa)	1647.6 ± 0.93	1635.2 ± 0.95	1623 ± 0.86	1610.9 ± 0.87
E_3_ (MPa)	1647.6 ± 0.93	1635.2 ± 0.96	1623 ± 0.86	1610.9 ± 0.84
G_12_ (MPa)	611.59 ± 0.23	608.38 ± 0.24	605.19 ± 0.23	602.02 ± 0.22
G_23_ (MPa)	611.57 ± 0.24	608.35 ± 0.25	605.15 ± 0.23	601.98 ± 0.22
G_31_ (MPa)	611.59 ± 0.23	608.38 ± 0.24	605.19 ± 0.22	602.02 ± 0.22
ʋ_12_	0.347 ± 2.13	0.344 ± 2.24	0.341 ± 2.11	0.338 ± 2.1
ʋ_13_	0.346 ± 2.13	0.343 ± 2.24	0.340 ± 2.13	0.338 ± 2.07
ʋ_23_	0.346 ± 2.24	0.343 ± 2.24	0.340 ± 2.17	0.337 ± 2.13
Density (g/cm^3^)	1.10 ± 2.23	1.11 ± 2.33	1.11 ± 2.21	1.11 ± 2.10

**Table 12:** Engineering constants obtained by the ACP model for carnauba–epoxy composite laminates.

Properties	Samples			
	EC10	EC20	EC30	EC40
Flexural Laminate Shear Stiffness (MPa)	611.59	608.38	605.19	602.02
Flexural Laminate Stiffness E_1_ (MPa)	1648.13	1636.24	1624.32	1612.37
Flexural Laminate Stiffness E_2_ (MPa)	1647.56	1635.23	1623.01	1610.87
Laminate Shear Stiffness (MPa)	611.59	608.38	605.19	602.02
Laminate Stiffness E_1_ (MPa)	1648.13	1636.24	1624.32	1612.37
Laminate Stiffness E_2_ (MPa)	1647.56	1635.23	1623.01	1610.87
Out-of-Plane Shear G_23_ (MPa)	509.64	506.96	504.29	501.64
Out-of-Plane Shear G_31_ (MPa)	509.65	506.98	504.32	501.64
Shear Correction Factor k_44_ (G_23_)	0.833	0.833	0.833	0.833
Shear Correction Factor k_55_ (G_31_)	0.833	0.833	0.833	0.833

**Table 13:** Comparison between residual velocities obtained experimentally and via numerical simulation.

**Samples**	**Experimental**		
	**V_i_ (m/s)**	**V_r_ (m/s)**	**E_abs_ (J)**
EC10	817.07	782.97	264.54
EC20	812.11	781.35	237.80
EC30	819.19	790.78	221.97
EC40	835.62	805.46	240.00
**Samples**	**Numerical Simulation**		
	**V_i_ (m/s)**	**V_r_(m/s)**	**E_abs_ (J)**
EC10	850.0	843.85	50.52
EC20	850.0	843.69	51.83
EC30	850.0	843.98	49.45
EC40	850.0	843.89	50.19

## Data Availability

The original contributions presented in the study are included in the article; further inquiries can be directed to the corresponding author.
